# Supplementary material for: Electrical spectroscopy of polaritonic nanoresonators
Source: Nat Commun. 2024 Oct 5;15:8635. doi: 10.1038/s41467-024-52838-w (PMC11452637; doi:10.1038/s41467-024-52838-w)
Supplement: Supplementary file 1 — Supplementary Information [file 41467_2024_52838_MOESM1_ESM.pdf]

Supplementary Information: Electrical Spectroscopy of Polaritonic Nanoresonators

Sebastián Castilla,[1] Hitesh Agarwal,[1] Ioannis Vangelidis,[2] Yuliy V. Bludov,[3,4] David Alcaraz Iranzo,[1] Adrià Grabulosa,[1] Matteo Ceccanti,[1] Mikhail I. Vasilevskiy,[3,4,5] Roshan Krishna Kumar,[1] Eli Janzen,[6] James H. Edgar,[6] Kenji Watanabe,[7] Takashi Taniguchi,[8] Nuno M.R. Peres,[3,4,5,9] Elefterios Lidorikis,[2,10] and Frank H.L. Koppens[1,11]

[1] *ICFO - Institut de Ciències Fotòniques, The Barcelona Institute of Science and Technology, Castelldefels (Barcelona) 08860, Spain*
[2] *Department of Materials Science and Engineering, University of Ioannina, 45110 Ioannina, Greece*
[3] *Centro de Física (CF-UM-UP), Universidade do Minho, Campus de Gualtar, P-4710-057 Braga, Portugal*
[4] *Departamento de Física, Universidade do Minho, Campus de Gualtar, P-4710-057 Braga, Portugal*
[5] *International Iberian Nanotechnology Laboratory (INL), 4715-330 Braga, Portugal*
[6] *Tim Taylor Department of Chemical Engineering, Kansas State University, Manhattan, KS, USA*
[7] *Research Center for Electronic and Optical Materials, National Institute for Materials Science, 1-1 Namiki, Tsukuba 305-0044, Japan*
[8] *Research Center for Materials Nanoarchitectonics, National Institute for Materials Science, 1-1 Namiki, Tsukuba 305-0044, Japan*
[9] *POLIMA—Center for Polariton-driven Light-Matter Interactions, University of Southern Denmark, Campusvej 55, DK-5230 Odense M, Denmark*
[10] *University Research Center of Ioannina (URCI), Institute of Materials Science and Computing, 45110 Ioannina, Greece*
[11] *ICREA - Institució Catalana de Recerca i Estudis Avançats, 08010 Barcelona, Spain*

SUPPLEMENTARY NOTE 1: DESCRIPTION OF THE DEVICES CHARACTERISTICS

We produced five devices in total. Owing to the different requirements, we use two device configurations depending on the type of measurements, by mainly varying the grating location, as shown in Fig. 1a in the main text. We fabricated three devices that are exclusively for FTIR and two others for photocurrent measurements. For FTIR, we required a large optically active area (∼30x30 µm^2) of the hBN-encapsulated graphene combined with metallic nanorods on top of the 2D stack for devices 1 and 4, as shown in Fig. 1a of the main text, Supplementary Fig. 1a-c, and the inset of Fig. 1c in the main text. This configuration provides an ideal situation for launching efficiently the polaritons[1,2] and achieving uniform gating across the graphene channel by using a Si backgate, which enhances the optical response of the polaritonic nanoresonators by obtaining the same low damping rate across the graphene channel with a uniform gating profile (see Supplementary Fig. 2c-d, which shows that the damping rate decreases with the increase of the Fermi level). However, owing to this configuration of a single backgate, we are not able to produce a proper graphene pn-junction, and thus, cannot perform efficient photodetection measurements.

One of the two main drawbacks of this platform is the highly challenging fabrication procedure to obtain a relatively large clean interface of the van der Waals heterostructures and homogeneous metallic nanorods across this large area, with the purpose of obtaining a decent signal-to-noise-ratio (SNR) in transmission measurements. In fact, even though achieving the minimum optically active area required, the SNR could still be low, as shown for device 4 results, in Supplementary Fig. 5. The second challenge is the metallic nanogratings arduous lift-off step, as shown in Supplementary Figure 1a, which sometimes requires sonication that harms the graphene and 2D stack quality, hence obtaining a quite low fabrication yield.

In order to obtain higher yield of fabrication for these devices, we change the device configuration by placing the gratings below the 2D stack (for devices 2, 3 and 5, see Figs. 1c, Supplementary Figures 1d and 24a). In fact, since the first fabrication steps are to pattern and evaporate the metallic gratings, we can sonicate the substrate to avoid any lift-off issues, thus achieving a high yield of fabrication of these nanostructures. Also this alternative device configuration allows us to pattern the metallic gratings of device 3 using Ga FIB (gallium focused ion beam), thus obtaining sharper metallic edges of the gratings and higher resolution of the features, as shown in Fig. 1b in the main text. In contrast to the devices 1 and 4, in devices 2, 3 and 5 we use the gratings with a two-fold purpose: 1) to provide enough momentum to launch the 2D polaritons and 2) to dope graphene by using them as a bottom split gate to create a graphene pn-junction, as shown in Fig. 2. For the photocurrent measurements, the device area can be smaller (e.g. device 2 has an area of 6 × 3 µm^2, see Supplementary Figure 1d) since we could get reasonable SNR in these measurements owing to the small photoactive area to produce a signal in the graphene pn-junction[3]. It is worth mentioning that although the grating gates create a graphene pn-junction for efficient photocurrent measurements, these nanostructures produce a non-uniform doping profile, as shown in the previously mentioned figures (Supplementary Fig, 2c-d). Therefore, we conclude that the ideal platform that accomplishes all the requirements to perform simultaneously both transmission and photocurrent measurements is highly challenging to fabricate and is out of the scope of this manuscript.

The 5 devices are listed in Supplementary Table I with their respective characteristics. We mainly vary the grating period (L is the period of the metallic rods that consists on the sum of the metallic width (w) and the gap between these rods (g)), substrate, gratings fabrication procedure (fabricated using EBL or FIB), hBN type (natural or enriched isotope) and the graphene channel area of each device.

**Sample**	**Grating location**	**Grating fabrication**	**Grating period ($L = w + g$)**	**Substrate**	**hBN type**	**Thickness hBN top / bottom**	**Channel area (length × width)**
Device 1	above 2D stack	EBL	150 = 100 + 50 nm	SiO_2	natural	13 / 4.5 nm	30 × 22 µm^2
Device 2	below 2D stack	FIB	75.5 = 38.5 + 37 nm	SiO_2	iso-B10	6.5 / 6 nm	6 × 3 µm^2
Device 3	below 2D stack	EBL	100 = 50 + 50 nm	CaF_2	natural	28 / 5 nm	24 × 23 µm^2
Device 4	above 2D stack	EBL	100 = 50 + 50 nm	SiO_2	natural	12.5 / 8 nm	30 × 25 µm^2
Device 5	below 2D stack	EBL	66 = 34 + 32 nm	SiO_2	natural	20 / 4 nm	12 × 5 µm^2

Supplementary Table I. Characteristics of the fabricated devices.

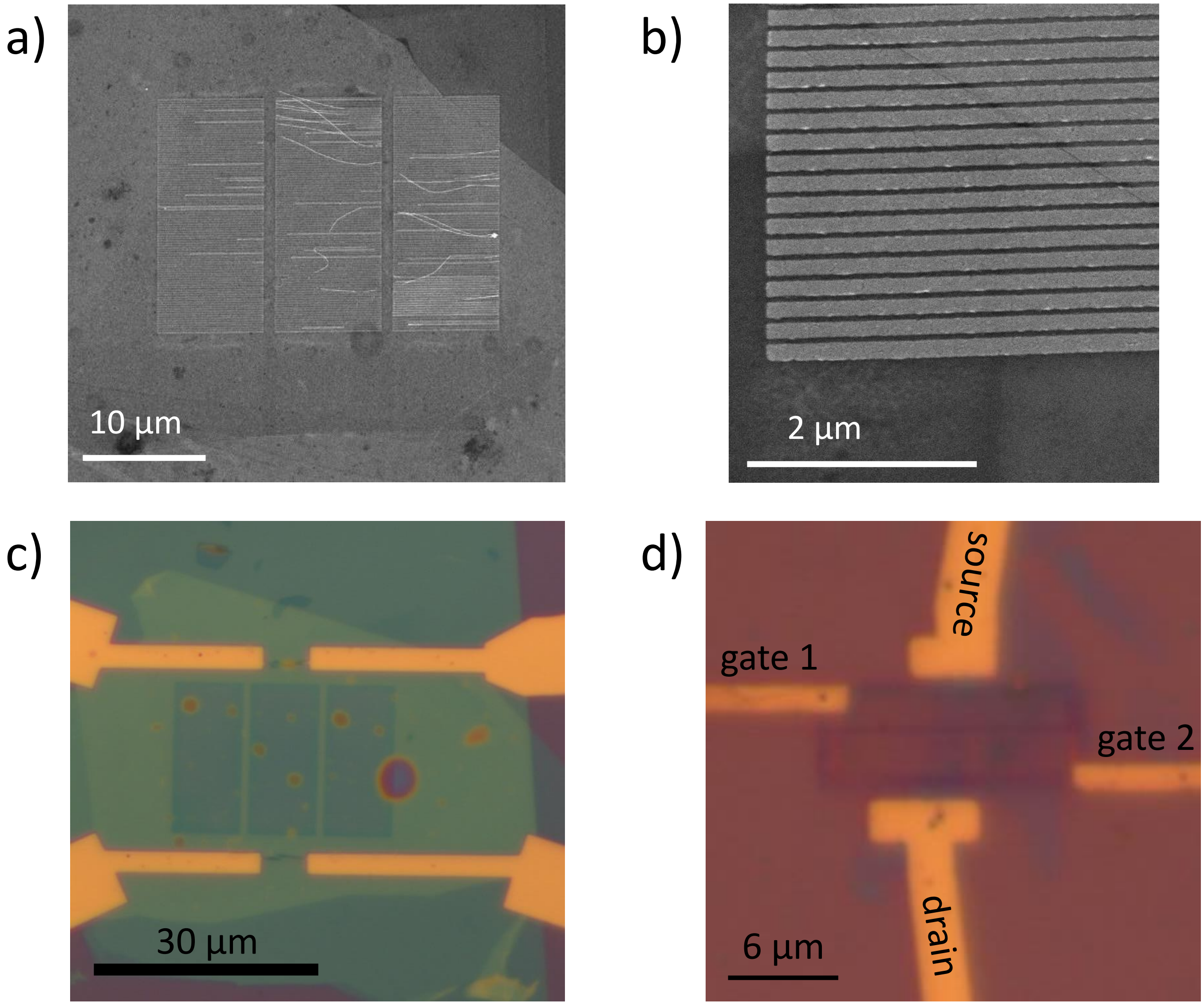

Supplementary Figure 1. **a)** SEM picture of the gratings on top of the 2D stack of device 1. **b)** Zoomed SEM image of the gratings in **a**. **c)** Optical image of device 4. **d)** Optical image of device 2.

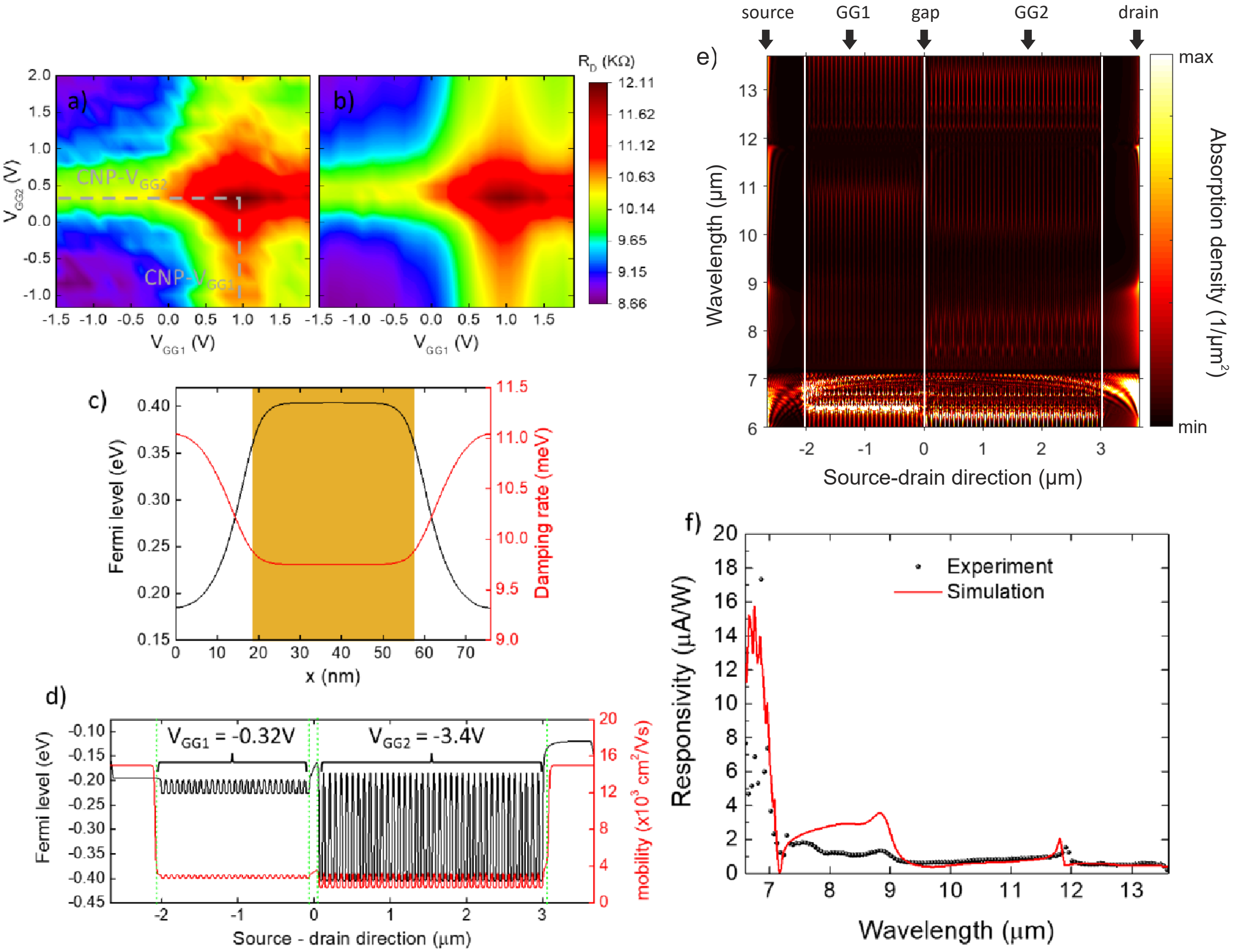

Supplementary Figure 2. **a)** Two-terminal experimental and **b)** fitted resistance as a function of the two grating gates voltages (GG1 and GG2) for Device 2. The resistance was fitted using the model described in ref. 3, yielding an average field effect mobility $<\mu>\sim$ 3,000 cm^2/Vs contact resistance R_C = 7.2 kΩ, residual doping of 11.3×10^{11} cm^{-2} at the graphene above GG1 and 6.8×10^{11} cm^{-2} at the graphene above GG2 and V_{CNP} of 0.92 V and 0.34 V for GG1 and GG2, respectively. **c)** Non-uniform electrostatic potential profile (black curve) and damping rate (red curve) in a grating period for $|V_{GG2}|$ = 3.4 V. The yellow shaded region indicates the position of the Au grating gate. The damping rate of the graphene was modeled as $\frac{\hbar}{\tau_{MSC}} = \frac{\hbar}{\gamma|E_F|}$ for the ungated regions near the contacts, while at the gated regions was modified to $\frac{\hbar}{\tau_{MSC}} = \hbar(\frac{1}{\gamma|E_F|} + \frac{v_F}{<A>})$, with $<A>$ the average length where inhomogeneity in the metal and gap width of the rods appears, to account for an extra scattering mechanism that arises from the device's geometry disorder. γ was estimated at 1500 fs/eV and $<A>= D$ with D the grating period. **d)** E_F (black curve) and μ (red curve) profile across the graphene channel for V_{GG1} = -0.32 V and V_{GG2} = - 3.4 V. Above GG1 and GG2 $<\mu>\sim$ 2,500 cm^2/Vs, while in the ungated region in the vicinity of the contacts μ = 15,000 cm^2/Vs. The mobility was modeled as $\mu = \frac{e\tau_{MSC}v_f^2}{|E_F|}$. **e)** Graphene absorption density (1/μm^2) of Device 2 as a function of wavelength across the source drain direction (see Fig. 1e in the main text for axis definition, where x = 0 is the center of the gap between the grating gates as indicated with the white vertical line). In panel **e**, we show the spatially resolved absorption spectrum in graphene across the channel in the $x-$direction at the graphene doping of panel **d**. We notice different absorption peaks in the GG2 region compared to the GG1 one, where spectrally appear additional absorption peaks outside the RBs range. Also, at the RBs, we observe change of the spectral shape of the absorption peak due to the graphene doping. The end of each gate is indicated with the outer white lines. The absorption contribution at the spatial edges of the device occurs at the ungated graphene region in the vicinity of the contacts (at the left and right corners of the plot) and spectrally located mainly at the RBs of hBN and SiO_2. **f)** Experimental (black dots) and theoretical (red solid line) spectral external responsivity of the device 2 for GG1 at -0.32 V and GG2 at -3.4 V. The typical incident power ranges from ∼1-15 mW and an irradiance of 33.6 mW/μm^2. The devices 2 and 3 achieve a similar responsivity in the order of tens of μA/W. The low responsivity of these devices relies on the non-optimized design to exploit efficiently the PTE effect as shown in other studies[3,4]. We point out that the non-uniform graphene doping profile adds more complexity to the photoresponse modeling (see panels **c-e**) since the absorption of the periodic structure of the two gated regions contribute to the photoresponse besides the absorption around the graphene pn-junction interface[3], in particular for small devices (device 2).

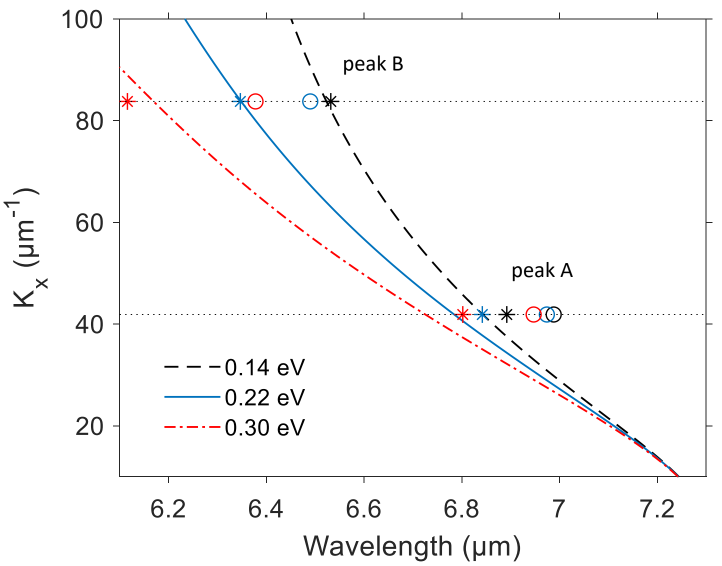

Supplementary Figure 3. Dispersion relation for device 1 of the hybridized plasmon phonon polariton modes at the upper RB of hBN for different Fermi levels (represented as lines in the legend). The two horizontal dashed lines correspond to the first and second diffraction order resonances launched by the metal rod array. The open dots and asterisks represent the experimental and theoretical values, which correspond to peaks A and B, as defined in Fig. 1c-d in the main text. The slight red shift of the experimental points with respect to the theoretical data is ascribed to the intrinsic doping of graphene.

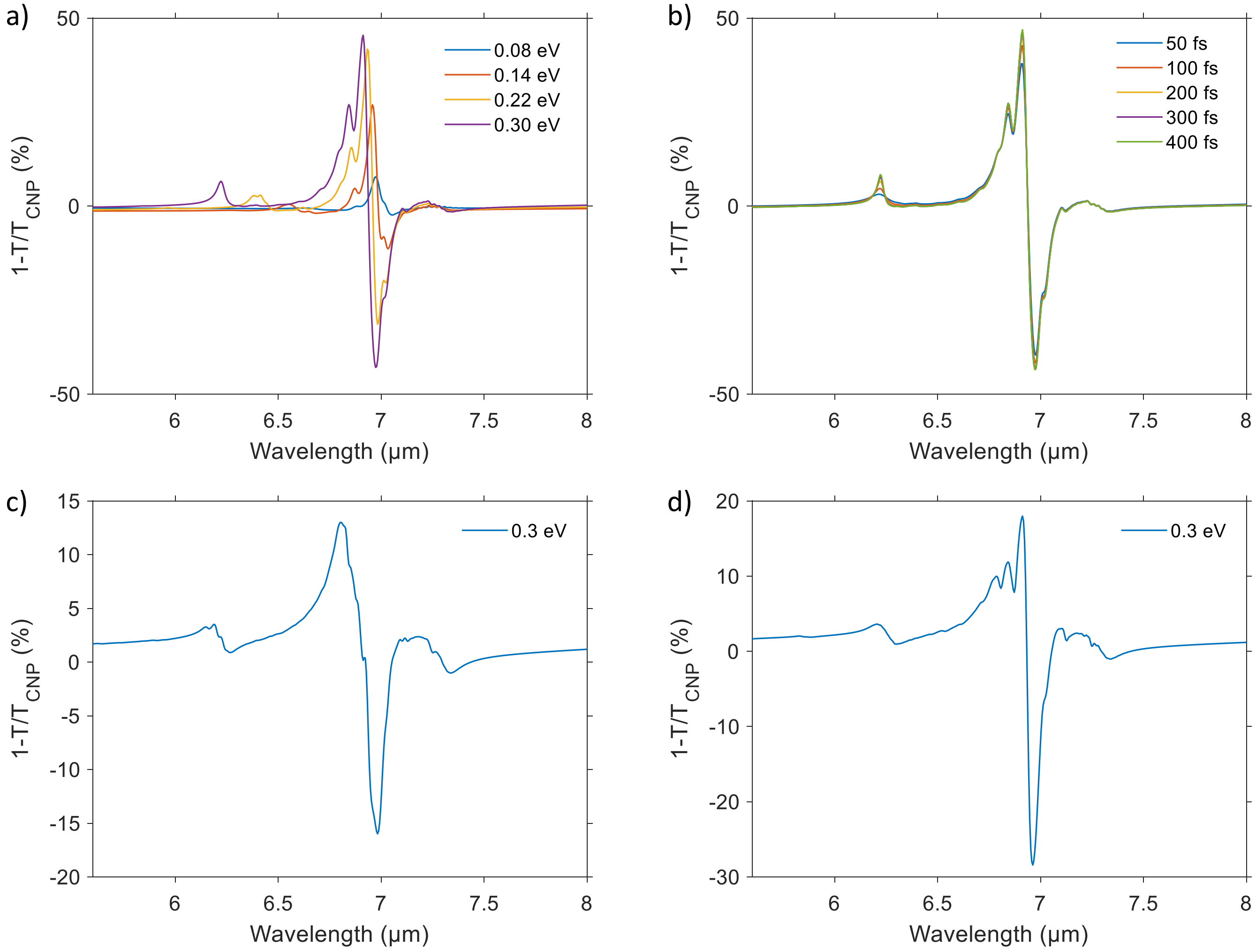

Supplementary Figure 4. Extinction simulations of device 1. **a)** Extinction spectra for different Fermi levels for a fixed scattering time of 200 fs. **b)** Extinction spectra for several scattering times for a fixed Fermi level of 0.3 eV. **c)** Extinction spectrum for a fixed Fermi level of 0.3 eV and scattering time of 200 fs. Here the extinction spectrum is calculated for a size distribution of the gap between the metals ranging from 30 to 70 nm. **d)** Same as **c** but the extinction spectrum is calculated for a size distribution of the metal width ranging from 80 to 120 nm. All the simulations consider a graphene mobility of 10,000 cm^2/Vs. The extinction spectra in **c** and **d** are calculated as follows: $E_d = \sum_{i=a_0-N/2}^{i=a_0+N/2} E_i f(i; a_0, \sigma^2)$, where E_d the extinction spectra in the disorder case, a_0 the central value of the metal width (gap width) distribution in **c** (**d**), N the extent of the distribution, E_i the extinction spectra for each i metal width (gap width) and f a probability function centered at a_0 with standard deviation $\sigma = 6$ nm.

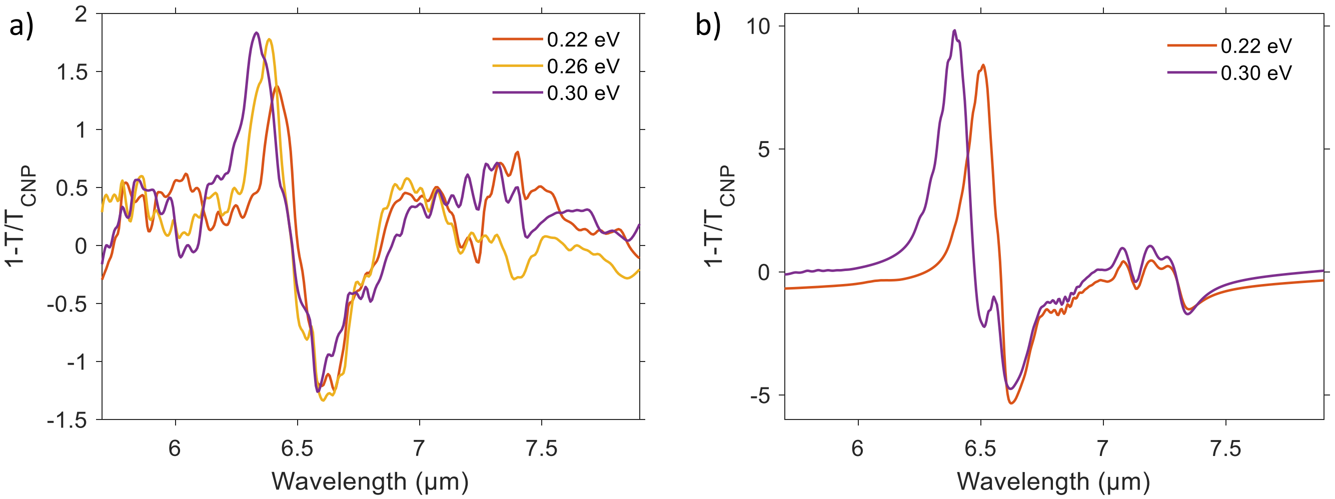

Supplementary Figure 5. Extinction measurements and simulations of device 4. **a)** Extinction (1-T/T_{CNP}) spectrum of device 4 measured using FTIR. The curves correspond to several Fermi levels, as indicated in the legend. Despite the low extinction values, we are able to probe the polaritonic resonance. **b)** Simulated extinction spectra of device 4 for several Fermi levels. We use a graphene mobility of 10,000 cm^2/Vs. As explained previously, we use a size distribution of the gap between the metals with a standard deviation $\sigma = 10$ nm.

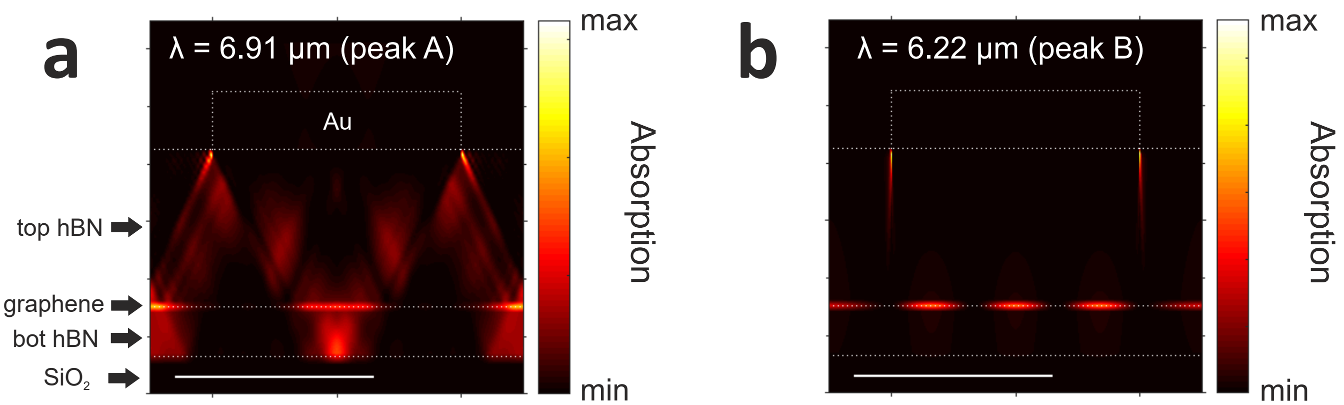

Supplementary Figure 6. Absorption profiles of transmission measurements of device 1. **a)** Cross section view of the simulated absorption of the 2D stack at $\lambda = 6.91$ μm (1447 cm^{-1}) for a graphene Fermi level of 0.3 eV. The white scale bar corresponds to 50 nm. **b)** Same as **f** but at $\lambda = 6.22$ μm (1607 cm^{-1}). **a** and **b** $y-$axis is not to scale for illustration.

For peak A shown in Fig. S6a, we observe that the hBN hyperbolic phonon polaritons (HPPs) are launched at the edges of the metallic gratings and propagate as ray-like waves through the hBN slab. Simultaneously, in the graphene layer plane, a plasmonic wave is observed that also constructively interfere with the HPPs. This behavior corresponds to the hybridize plasmon-phonon polaritons as shown in near-field experiments via s-SNOM[5,6]. This is in agreement with the relatively small wavenumber shift (≈ 20 cm^{-1}) with the Fermi level as shown in Fig. 1c in the main text and is ascribed to the phonon-like nature of this polariton.[5,6] On the other hand, for peak B (Fig. S6b), we don't observe a clear hybridization since the graphene plasmon mode resonates at its plane without hBN HPPs interferences. This might be due to the fact that this peak is spectrally located at the edge of the upper RB, where the HPPs angles become more steep (close to $\sim$90º).[7] We point out that peak A shows a narrower linewidth (see Fig. 1c-d in the main text) and higher extinction value ascribed to the low-loss nature of this hybridized polariton[5,6] in comparison with the peak B.

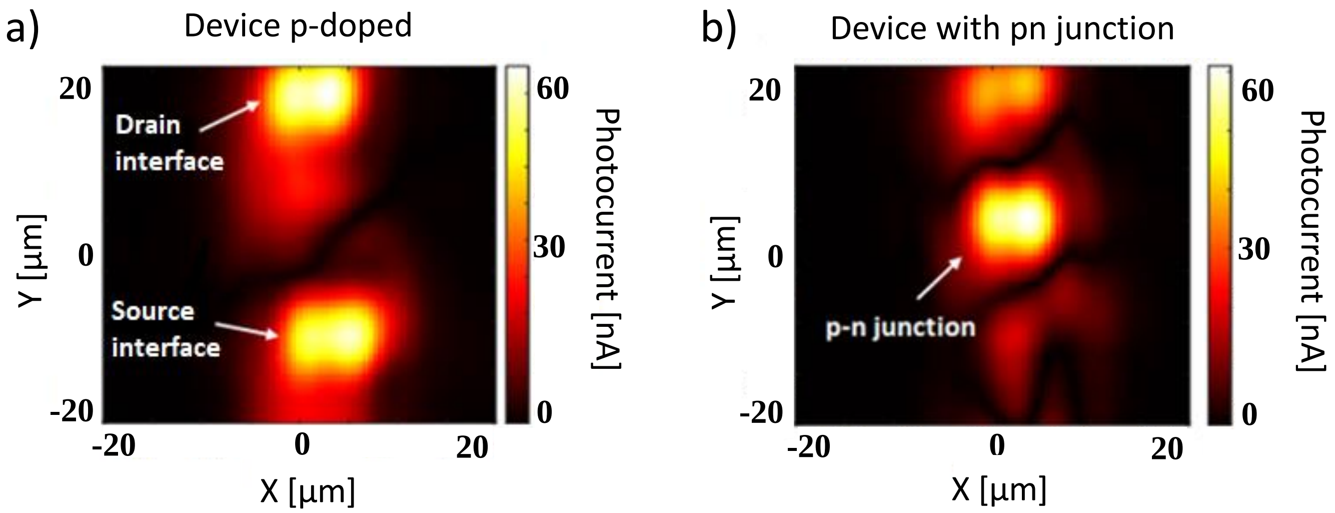

Supplementary Figure 7. Scanning photocurrent map as function of the x and y motorized stage position at incident wavelength of 6.6 µm for **a)** uniformly p-doped graphene channel with both gates (GG1 and GG2) set at -0.2 V and for **b)** pn-junction graphene channel configuration with GG1 at 0.4 V and GG2 at -0.25 V. The measurements correspond device 3. The photocurrent signal corresponds to the absolute value of the signal without considering the sign change of the photocurrent in vicinity to the metal electrodes.

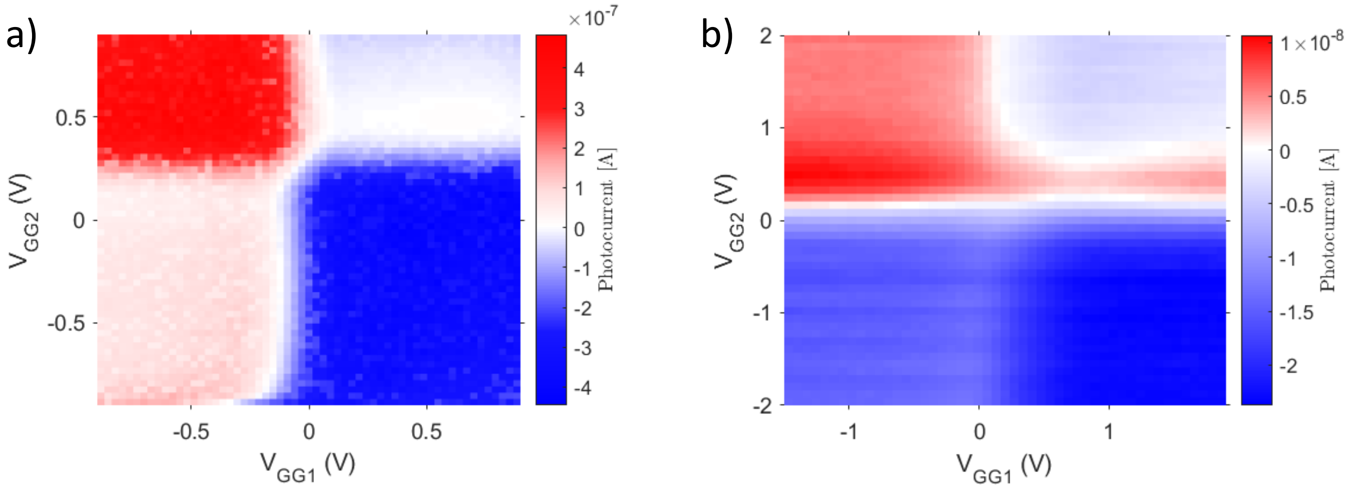

Supplementary Figure 8. Photocurrent map as a function of the grating gate voltages (GG1 and GG2) for **a)** device 3 at incident wavelength of 7 µm and **b)** device 2 at incident wavelength of 7.75 µm.

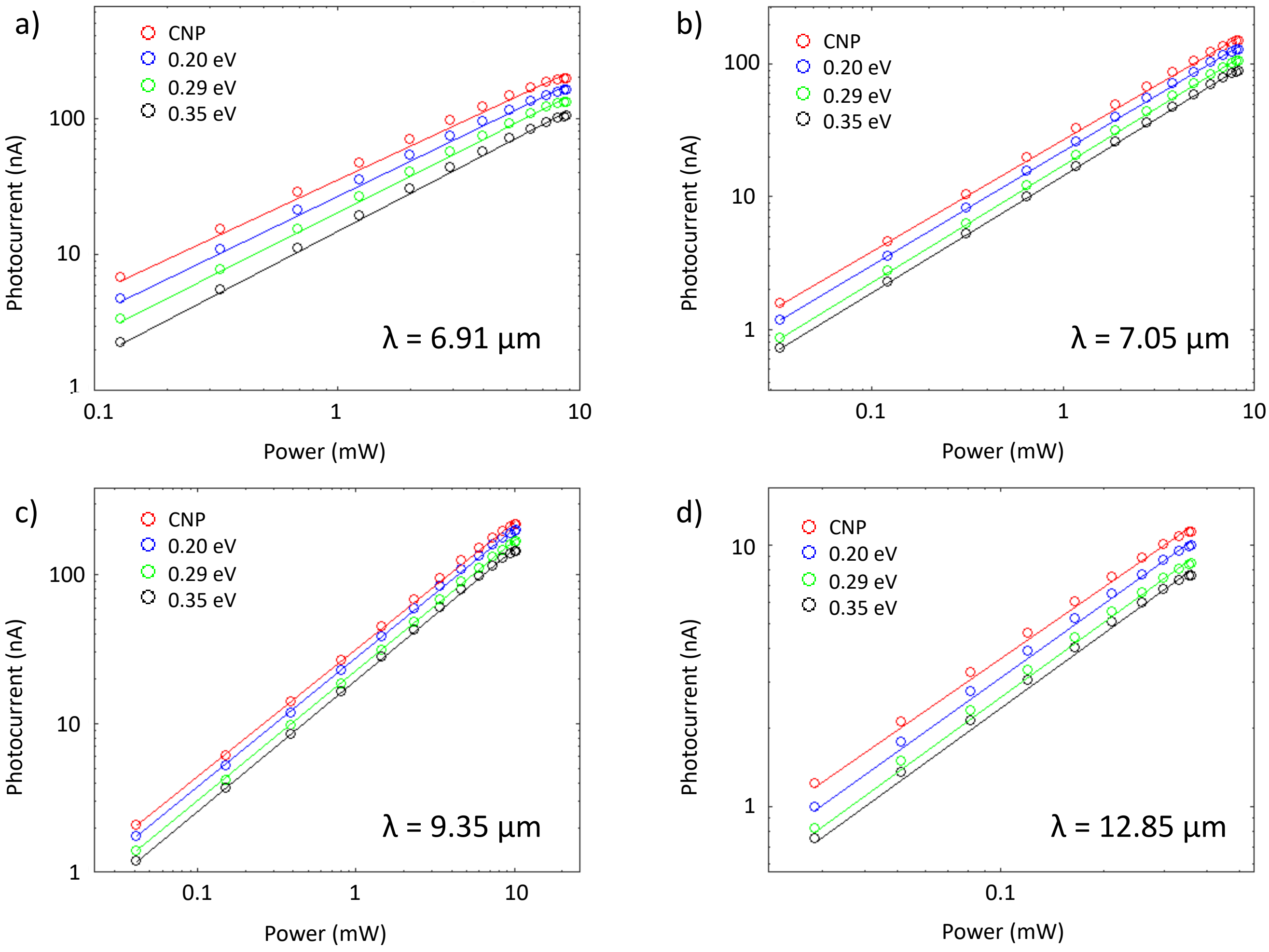

Supplementary Figure 9. Power dependence of the photoresponse of device 3. Photocurrent as a function of the incident power for several wavelengths: **a)** at 6.91 µm, **b)** at 7.05 µm, **c)** at 9.35 µm and **d)** at 12.85 µm. For all the cases we plot for different gate voltages of GG2 that 0 V corresponds to the CNP and -2.4 V to a graphene Fermi level of 0.35 eV. GG1 remains fixed at 0.5 V. The opened circles represent to the experimental points and the lines to the fit that show a linear dependence. This behavior corresponds to the weak heating regime as shown in previous studies.[3,8]

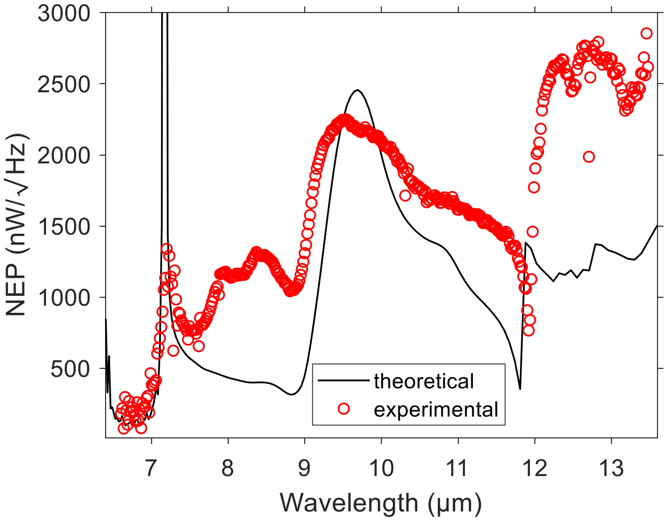

Supplementary Figure 10. Theoretical (black solid line) and experimental (red open circles) noise equivalent power (NEP) spectrum of device 2. Due to the zero-bias operation, we consider the Johnson noise as the main contribution of the noise in the graphene channel.[3,9] The resistance value of 8.5 kOhm corresponds to the gates' configuration of GG1 at -0.32 V and GG2 at -3.4 V. The minimum NEP corresponds to 77 and 94 nW/$\sqrt{Hz}$ for the experimental and theoretical respectively at ∼6.64 µm.

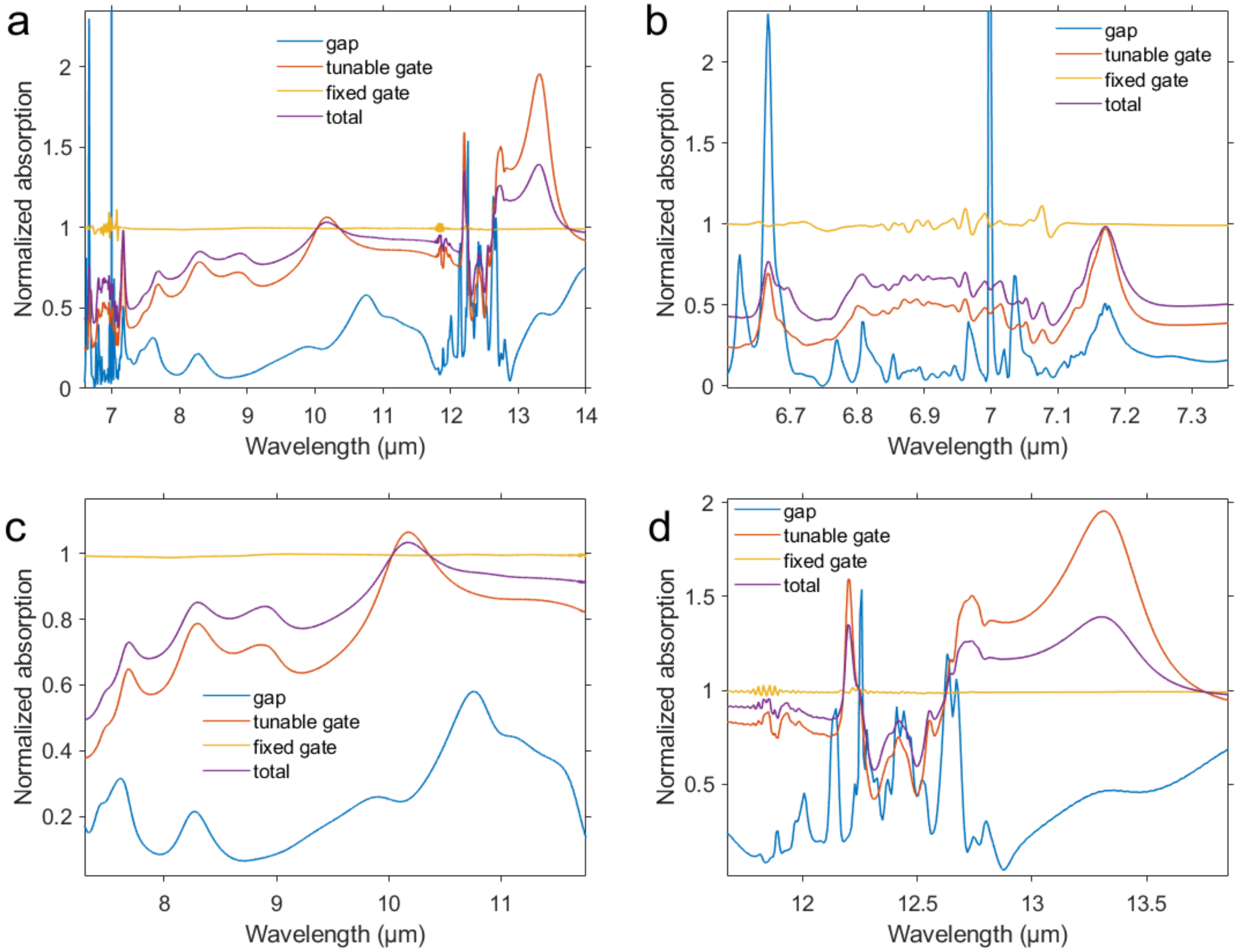

Supplementary Figure 11. Simulated graphene absorption spectrum at 0.41 eV normalized to the spectrum at CNP of device 2 for **a)** the experimentally measured wavelength range, **b)** upper RB range, **c)** between RBs and **d)** for lower RB range. We plot the different contributions of the graphene absorption in the each region of the graphene channel such as the absorption spectrum of the region above the grating gate 2 (GG2) that corresponds to the tunable gate normalized the spectrum at the CNP of that region, the absorption at the region above the gap of the gates normalized to the spectrum of the CNP of the gap region, and the total absorption, which the latter consists on the integrated absorption across the whole graphene channel including all the regions. The region above the grating gate 1 (GG1) that has a fixed Fermi energy (0.21 eV) is normalized to the same spectrum in order to verify the effect of the tunable gate, which we observe that the absorption above the GG1 region is not affected by the change of Fermi energy of GG2 region.

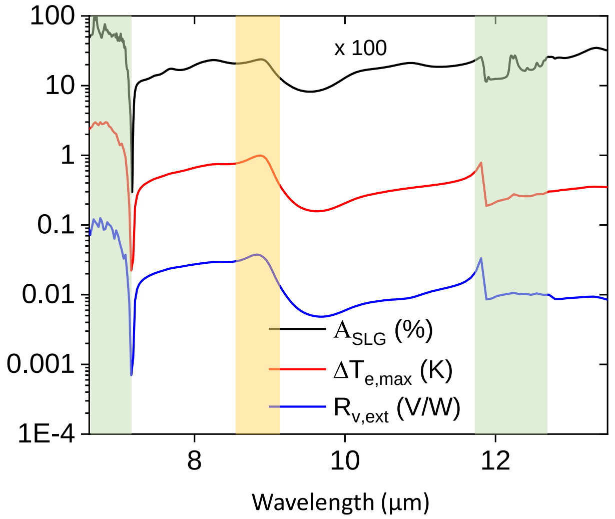

Supplementary Figure 12. **a)** Theoretical calculations of the absorption (in percentage), maximum value of the electronic temperature across the graphene channel (in K) and responsivity (in units of V/W) that share the y-axis for different units, as mentioned, respectively, which are plotted as a function of the wavelength. We observe that these three variables can be related to each other, as demonstrated in ref. 3. The highlighted spectral regions correspond to the RBs mentioned in the main text. For the input powers used in our experiments (1-10 mW and irradiance of 33.6 mW/μm^2), the estimated rise in electronic temperature ($\Delta T_e = T_{el}$-T_L, where T_L is the lattice temperature) is minimal, ranging from ∼0.1 K to ∼3 K (weak heating regime)[4,8]. Consequently, both the heat capacity and thermal conductivity can be considered temperature-independent and thus absorption-independent, given that the electron temperature rise is linked to absorption.[3,10] Therefore, the cooling length remains unaffected by the spectral dependence of absorption. Our simulations assume a spectrally independent value of 1.5 ps well within this range, resulting in good agreement between the measured and simulated spectral responsivity.[8,10] In our study, the carrier density is spectrally independent due to the small electronic temperature as mentioned previously and low photon energy at mid- and long-wave infrared frequencies (∼0.1 eV).

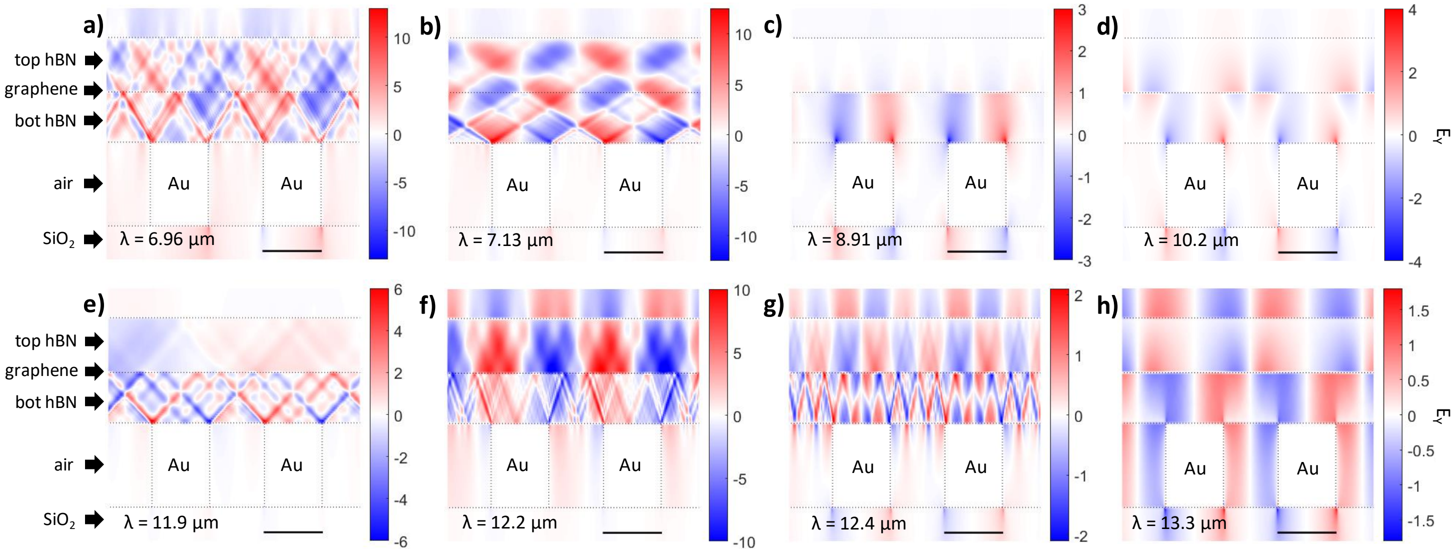

Supplementary Figure 13. Cross-sectional view of the y-component of the electric field normalized to the incident one across a region containing two metal nanorods for illustration. The Z direction (y-axis in graphs) and X direction (x-axis in the graphs) are defined in Fig. 1e in the main text. The black scale bar corresponds to 40 nm. The calculations correspond to a non-uniform graphene Fermi level with a value of 0.4 eV above the metal at **a)** wavelength 6.96 μm, **b)** 7.13 μm, **c)** 8.91 μm, **d)** 10.2 μm, **e)** 11.9 μm, **f)** 12.2 μm, **g)** 12.4 μm, **h)** 13.3 μm corresponding to peak 1, 2, 3, 4, 5, 6, 7 and 8 respectively labelled in Fig. 2a in the main text.

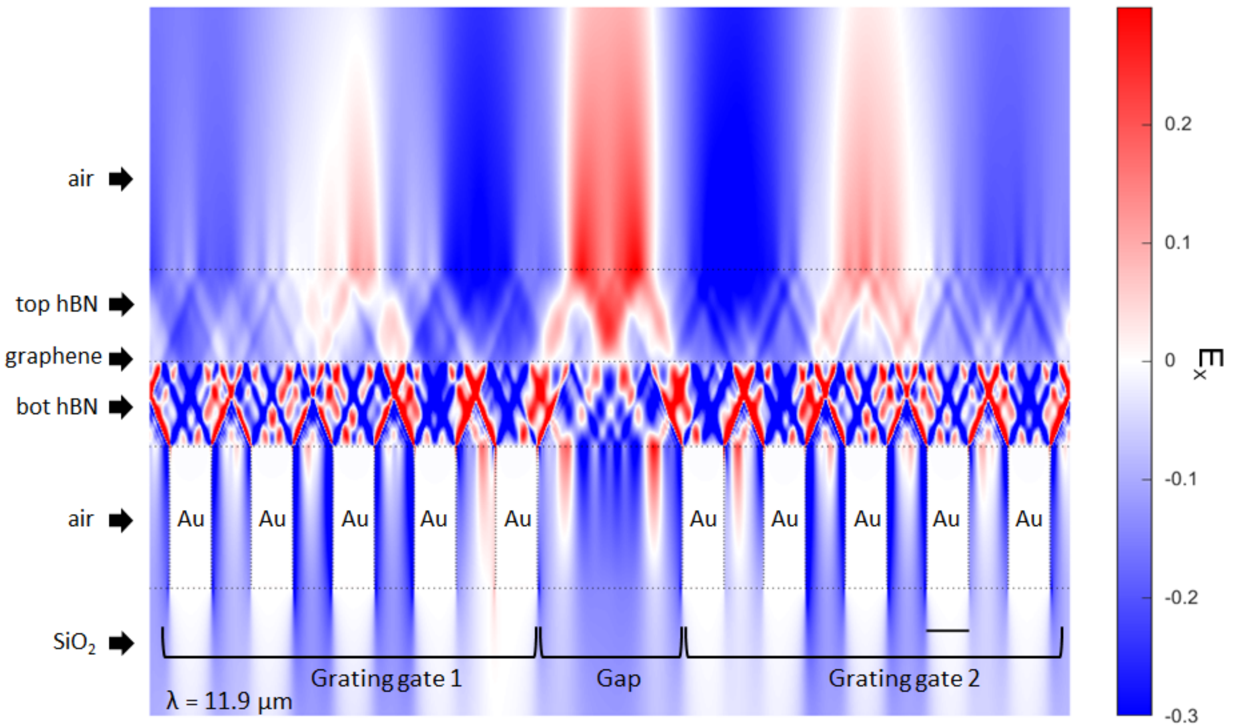

Supplementary Figure 14. Extended cross-sectional view of the $x-$component of electric field at $\lambda = 11.93$ µm, including the grating gates 1 and 2 and the gap between them. The black scale bar corresponds to 40 nm. Notably, peak 5 of the Fig. 2 in the main text presents a peculiar resonance by meeting the condition of $k_{\mathrm{eff}} = \pi/D$, which is interpreted as a defect mode with the diffraction order $n = 1/2$ owing to the broken symmetry of the grating.

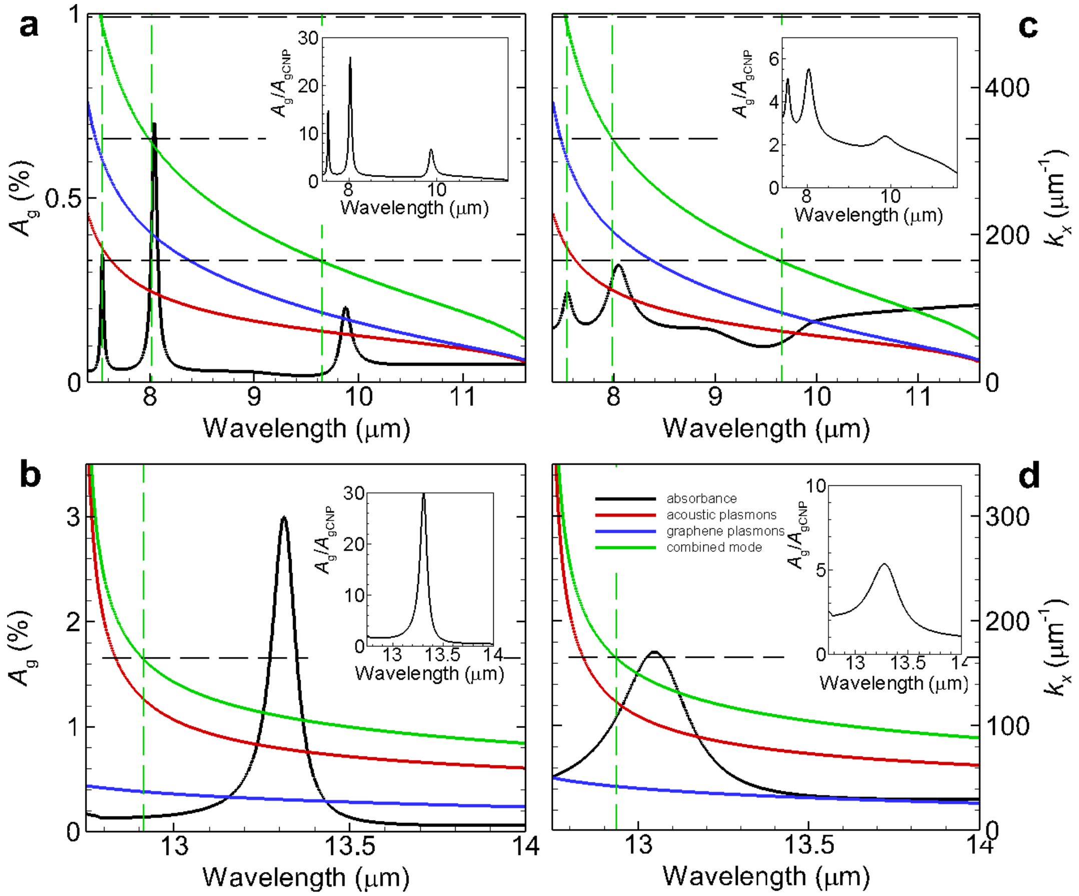

Supplementary Figure 15. **a-d)** Theoretically calculated graphene absorbance for the device 2 (black solid lines, values correspond to left vertical axis) and eigenmode wavevector k_x (red, blue and green lines for acoustic plasmons, graphene plasmons and combined modes, respectively, values correspond to right vertical axis) versus vacuum wavelength λ (horizontal axis). In each panel **a-d**, the inset show the graphene absorbance, normalized to the one at the charge neutrality point. All the absorbance are calculated using the Drude model of graphene's conductivity and for the cases of high relaxation time τ (panels **a** and **b**), defined by Eqs. 13 and 14 without the v_F/D term (see Supplementary Note 3, section B) and low relaxation time (panels **c** and **d**, defined by Eq. 14). In panels **a-d**, the lattice vectors $k_x = 4\pi n/D$, with D as the grating period (containing the gap between the metal rods plus the metal width) and $(n = 1, 2, \dots)$ are depicted by black dashed horizontal lines (right vertical axis), while their crossing points with combined mode dispersion curves are depicted by green dashed vertical lines.

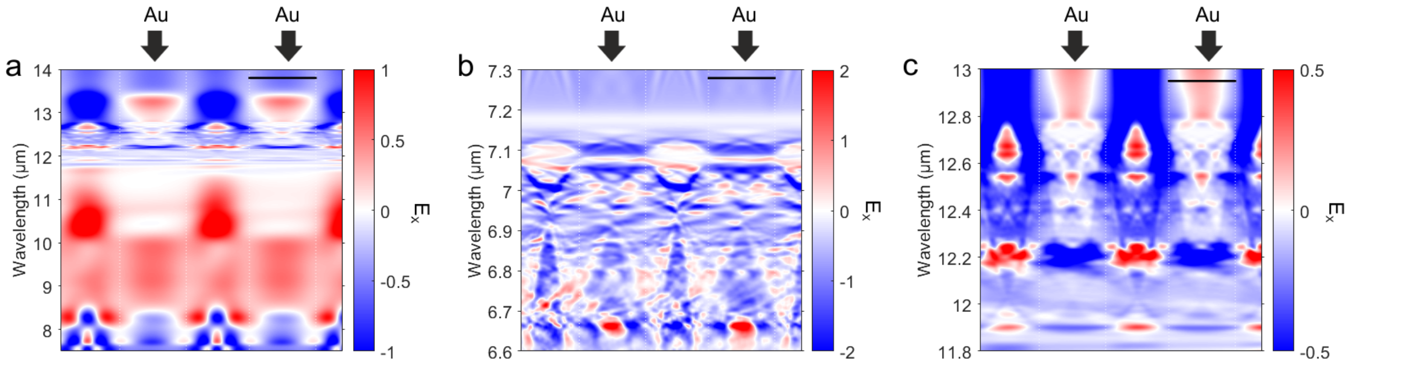

Supplementary Figure 16. **a)** Electric field intensity ($x-$component) map as a function of the incident wavelength and position along the source-drain direction containing two metal nanorods on the GG2 indicated in black arrows. The Fermi level is 0.41 eV. The black scale bar corresponds to 40 nm.

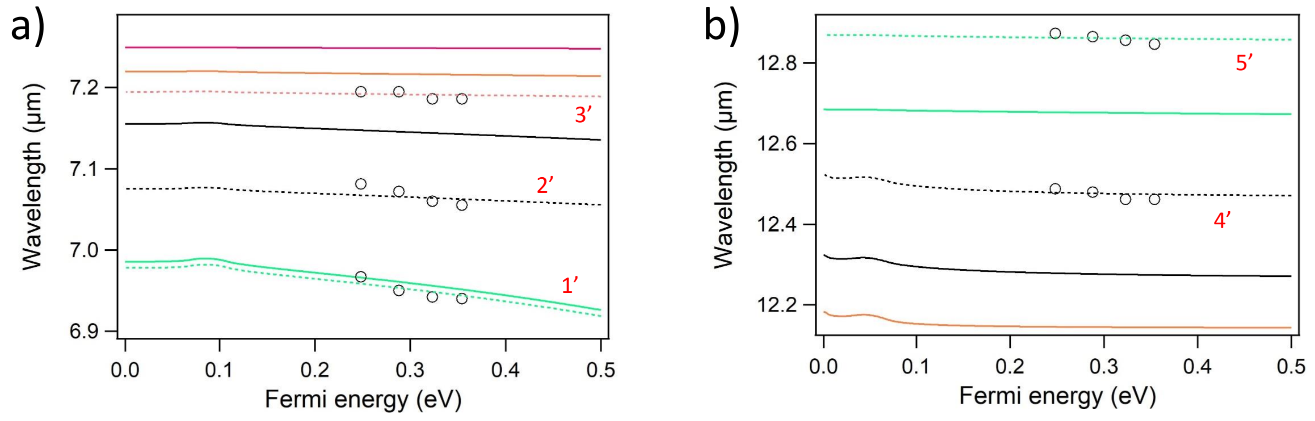

Supplementary Figure 17. Hybridized polaritons spectral peak position as a function of the Fermi energy for the **a)** upper and **b)** lower hBN RB. The open circles represent the experimental points found in Fig. 3. The solid lines are theoretical calculations and the dashed lines are the same but with a spectral offset to overlap the spectral shift respect to the experimental points. The colors of the lines represent the mode order. We observe that the spectral position of the peaks evolve in a sublinear manner according to $\sqrt{E_F}$, according to peak wavenumber $\propto \sqrt{E_F}^{0.01}$

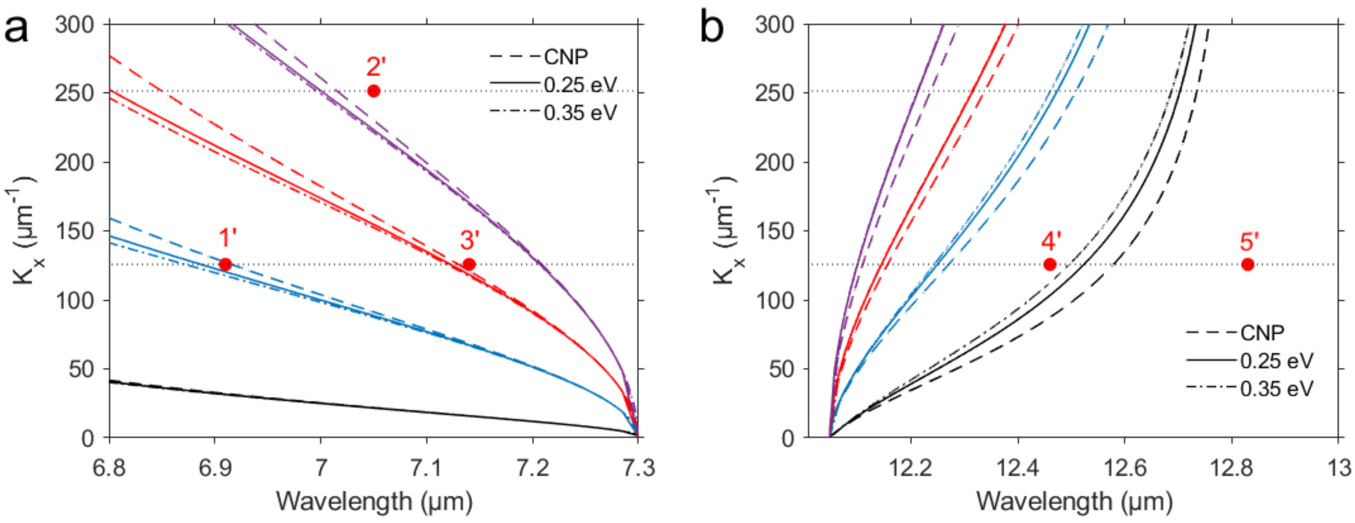

Supplementary Figure 18. **a)** Dispersion relation for device 3 of the hybridized plasmon phonon polariton modes at the upper RB of hBN. The two horizontal dashed lines correspond to the first and second diffraction order resonances launched by the metal rod array. The marked red dots represent the experimental values, which the numeric labels are defined in Fig. 3a in the main text. The graphene Fermi level is 0.35 eV. **b)** Same as panel **a** but at the lower RB spectral range.

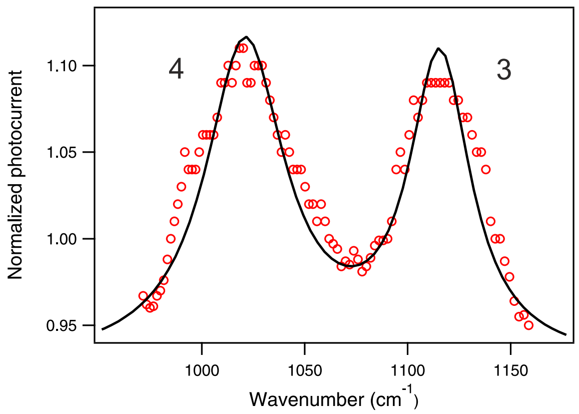

Supplementary Figure 19. Fit of polaritonic peaks of the normalized photocurrent. The fit corresponds to peaks 3 and 4 of device 2 shown in Figure 2a of the main text. We use a Lorentzian fit (black line) and we obtain a FWHM of 35.9 and 46.8 at 1115.6 cm^{-1} (8.96 µm) and 1021.1 cm^{-1} (9.79 µm) for peak 3 and 4, respectively (data shown in red open circles). By dividing the peak's wavenumber (wavelength) central position value by its FWHM, we obtain Q factors of 31 and 22 for peaks 3 and 4, respectively.

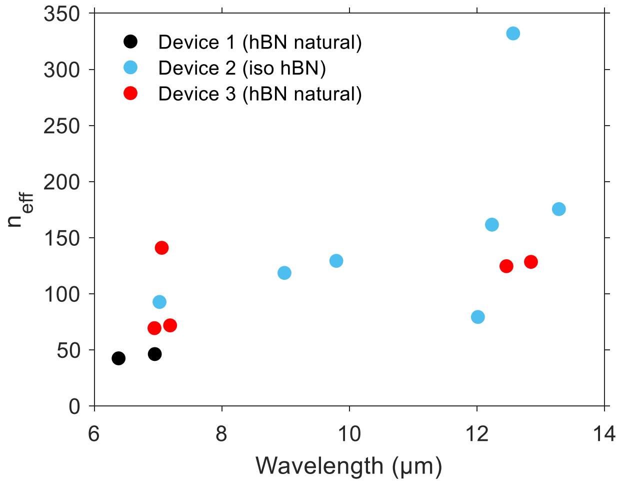

Supplementary Figure 20. Effective refractive index (n_{eff}) spectrum of the measured 2D polaritonic nanoresonators. The effective refractive index is defined as $n_{eff} \simeq k_p/k_{in}$,[11] where $k_p = 2\pi m/period$ is the polariton momentum, $k_{in} = 2\pi/\lambda_0$ is the incident wave momentum and m is the order of the resonance determined from the dispersion relations shown in previous graphs.

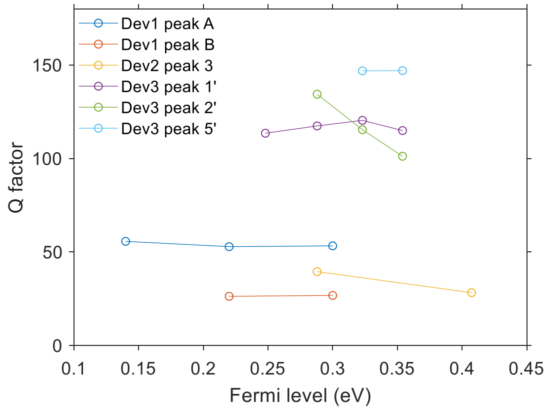

Supplementary Figure 21. Q factor values of the peaks observed in the devices 1-3 spectra as a function of the graphene Fermi level. We observe no significant changes of the Q factor when increasing the Fermi level in agreement with previous optical spectroscopy studies[12,13].

SUPPLEMENTARY NOTE 2: SIGNAL-TO-NOISE RATIO (SNR) OF FTIR AND PHOTOCURRENT MEASUREMENTS.

SNR of FTIR measurements

The signal-to-noise ratio (SNR) for FTIR measurements is the ratio of the height of an extinction peak to the noise in a particular spectral region. Usually, a reference spectrum is taken with an aperture size determined by the device area and with the graphene doping at the charge neutrality point. The subsequent spectra are taken at the same location but at different gate voltages to increase the graphene doping. The typical noise level in the upper RB spectral region is approximately ±1-2% in extinction and becomes significantly higher around the lower RB spectral region, as shown in the plots below.

Typical FTIR spectra require a smoothing procedure of the data consisting of a moving-average filter. This smoothing allows us to unveil hidden polaritonic peaks at the noise level, as in the case of device 4, as shown in Supplementary Figure 22. We point out that a smoothing procedure is necessary, despite the FTIR software performing an average step of repetitive scans (usually 5 to 50 scans) taken at a particular gate voltage.

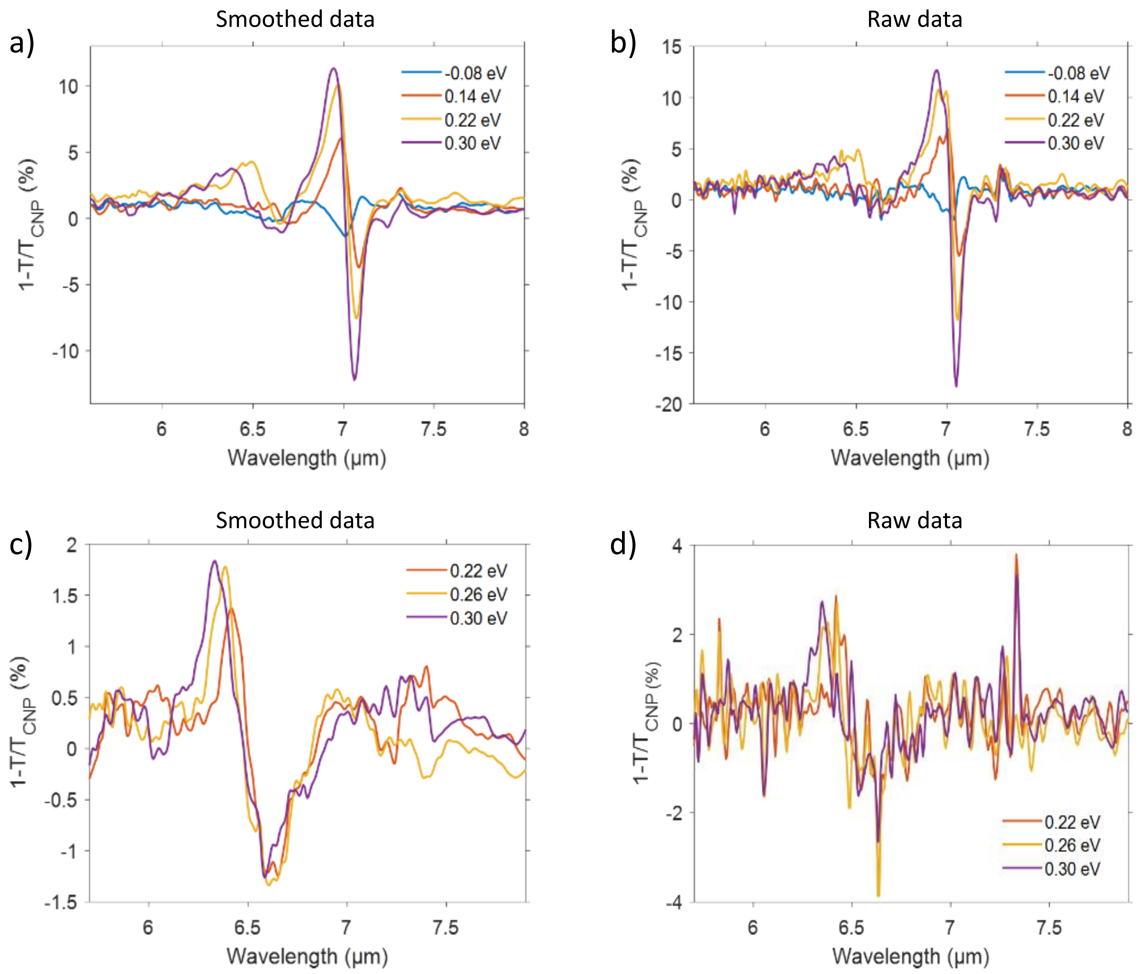

Supplementary Figure 22. **a)** and **c)** smoothed data of the extinction measurements of devices 1 and 4, respectively. **b)** and **d)** raw data of the extinction measurements of devices 1 and 4, respectively. For the smoothing procedure, we use a moving-average filter.

We note that the signal above 10 μm becomes very noisy, as shown in Supplementary Figure 23, containing a wider range of the spectrum. This increase in noise is probably due to the spectral limitations of the MCT detector, which at higher wavelengths, the detectivity and sensitivity drop significantly. Additionally, between 8 and 10 μm, the SiO_2 transversal optical phonon of the substrate is present and strongly absorbs mid-infrared light; hence,

the MCT detector receives a very small signal, and the SNR drops significantly. Owing to this large noise, we show a narrower spectral range that clearly shows polaritonic resonances, as shown in Figure 1 and Supplementary Figure 5.

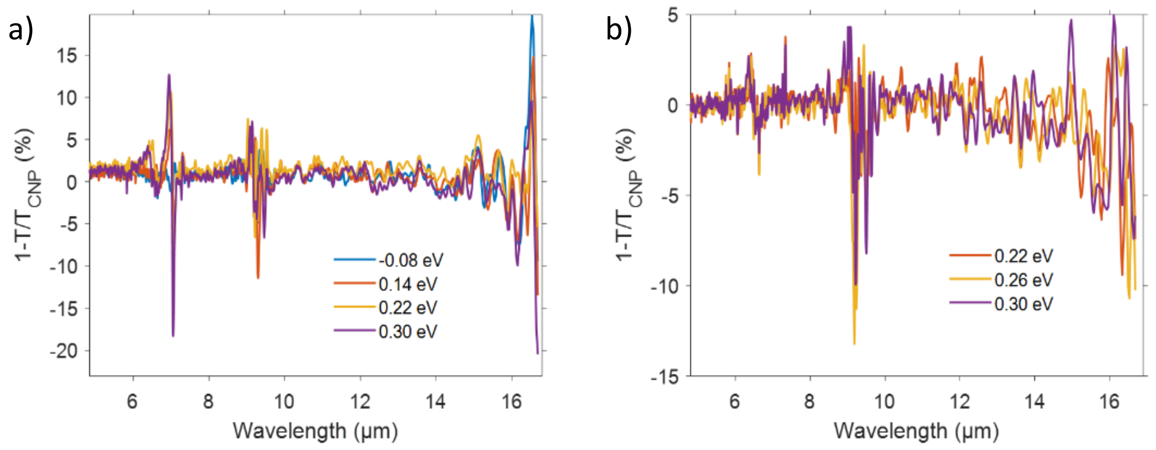

Supplementary Figure 23. Raw data of the extinction spectra, including the full wavelength range for devices **a)** 1 and **b)** 4.

To obtain higher spatial resolution, particularly for small samples, the aperture size must be reduced. However, increasing the spatial resolution results in a trade-off that leads to a decrease in SNR.[14] This is because a smaller aperture limits the amount of mid-infrared light that impinges on the MCT detector, which becomes even more challenging at higher wavelengths. Consequently, the MCT produces a lower signal and a significant decrease in the SNR. Therefore, for device 5, the aperture must be reduced to a very small area of approximately 12×5 μm^2, which leads to an SNR of 1, as shown in the Supplementary Figure 24. Although the electrical tunability of the resistance was achieved by simultaneously changing the gate voltage of the grating gates or silicon backgate (see Supplementary Figures 24e-f), the optical response did not show any clear polaritonic fingerprints, as shown in Supplementary Figure 24d. We note that the grating gates change the total graphene channel resistance less because of the small area covered by these gates with respect to the entire graphene channel, as shown in Supplementary Figure 24a. In addition, by performing RCWA simulations, we show that device 5 should show an extinction signal above 5% in the investigated wavelength range. The extinction values of device 5 are similar to those expected for device 2, as shown in Supplementary Figure 25.

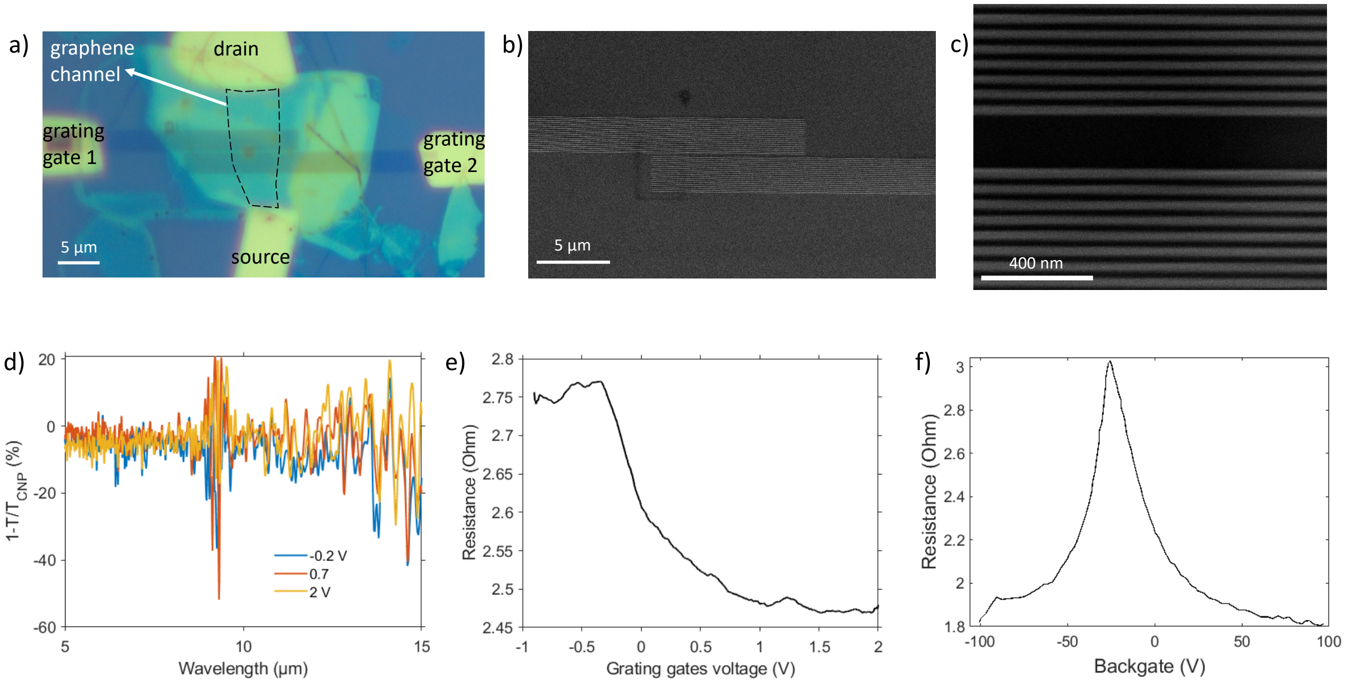

Supplementary Figure 24. **a)** Optical image of device 5. **b)** SEM image of the grating gates of device 5. **c)** Same as b but for a magnified region in the gap between the grating gates. **d)** Raw data of extinction spectra of device 5 for several voltages of the grating gates. **e)** Resistance as a function of grating gates voltage. The two grating gates are connected and swept at the same voltage. The CNP is located at -0.4 V. **f)** Resistance as a function of the silicon backgate.

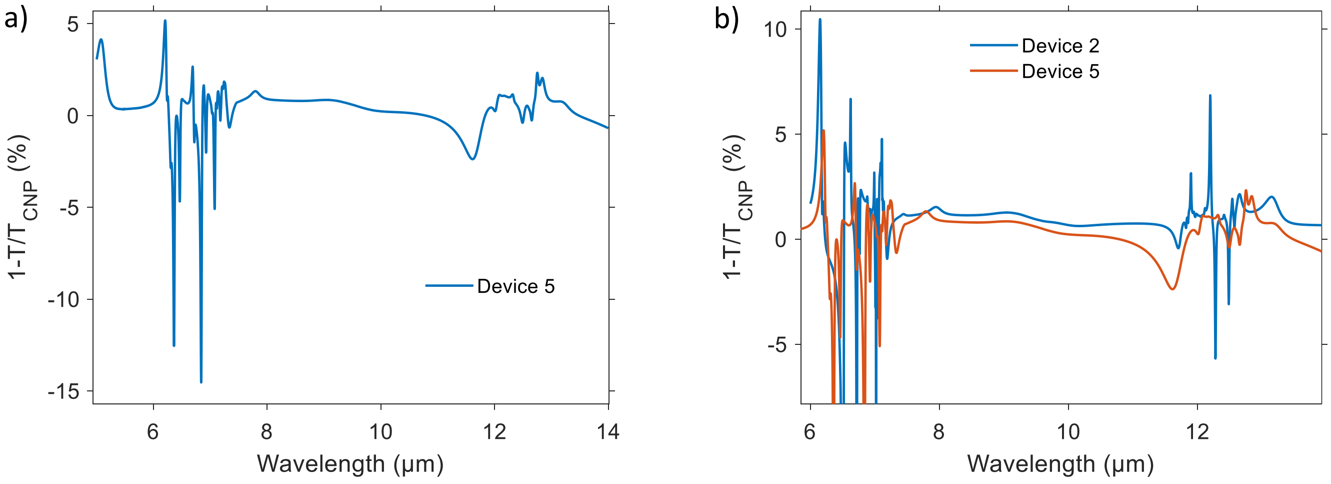

Supplementary Figure 25. **a)** Extinction spectrum of device 5 simulated using RCWA. We consider a periodic structure. The Fermi energy is 0.43 eV. **b)** Same as panel a but including the simulated extinction spectrum of device 2 for comparison. The simulated extinction of device 2 is calculated following the same procedure as that in panel a but with a Fermi energy of 0.41 eV.

We perform a complementary analysis to investigate the expected FTIR spectral signal of devices 2 and 5 and the impact of the noise levels. For this purpose, we employ the following procedure. We simulate the optical response of device 2 using the full-vector 2D finite-difference time-domain (FDTD) method by modeling the entire device structure in the lateral electrode-to-electrode direction. As the light source, we used a highly focused beam generated by a thin lens with a numerical aperture (NA) of 0.5, matching the experimental conditions. The calculated optical transmission (normalized to the CNP transmission) of the device for a specific combination of gate voltages (the same ones used to produce the black lines (corresponding to the highest doping) in Fig. 2a and 2b in the main text) is shown in Supplementary Figure 26a. All eight peaks identified in Figs. 2a and 2b of the main text are also observed in the FTIR spectrum. However, these calculations do not account for noise sources and thus provide an ideal FTIR signal.

To simulate a more realistic experimental signal containing noise, we artificially introduced white noise (i.e., frequency-independent) with three characteristic spans of ±1% (Supplementary Figure 26b), ±2.5% (Supplementary Figure 26c), and ±5% (Supplementary Figure 26d) using a commercial Mersenne Twister pseudorandom number generator (PRNG).[15] A ±1% span for the noise signal has been observed in the experimental FTIR signal of the large device 1 (active area of 660 μm^2), whereas the ±2.5% and ±5% noise spans are much closer to the experimental transmission spectrum of the much smaller device 5 (active area of 60 μm^2), which has similar dimensions to device 2. We observe that with the introduction of noise, even for the ±1% span noise signal, all polaritonic peaks outside the RBs are obscured. Simultaneously, the RB details are barely visible only at the smallest noise level and are completely hidden in the ±5% noise case. In Supplementary Fig. 26e, we show the extinction spectrum of device 5 calculated previously with RCWA (see Supplementary Fig. 25a), and we introduce frequency-dependent noise that increases exponentially at higher wavelengths, which starts at ±5% at 5-6 μm. We note that the polaritonic resonances are hidden within the noise, which resembles the experimental data shown in Supplementary Fig. 24d. This indicates the poor performance of FTIR signals in small devices and the difficulty in comparing such spectra with those obtained from electrical spectroscopy.

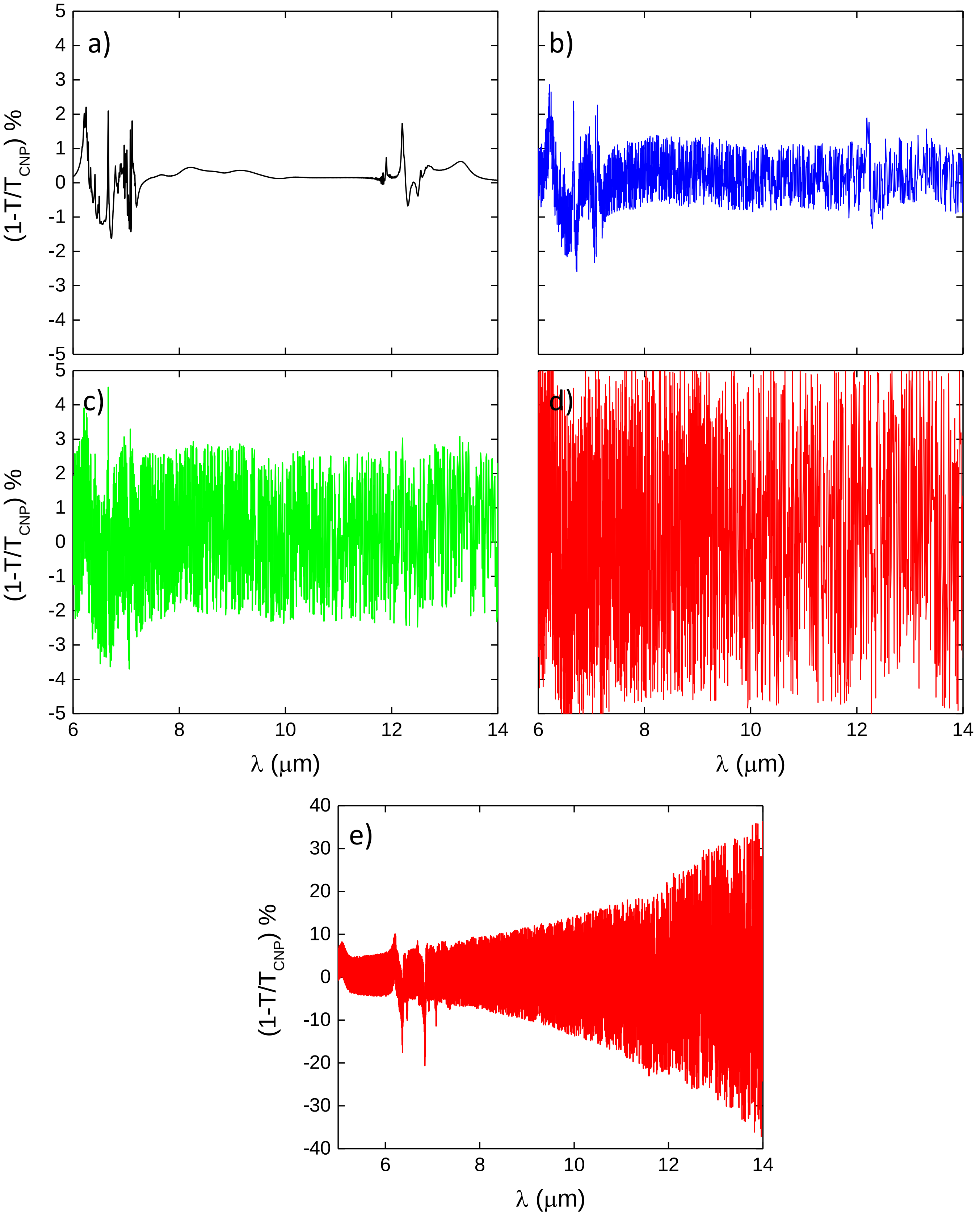

Supplementary Figure 26. **a)** Extinction spectrum of device 2 simulated using FDTD. The full structure of the device, including electrodes, was considered. **b)** Same as panel a but with white noise (frequency-independent) introduced in the spectrum of ±1%, **c)** ±2%, and **d)** ±5%. **e)** Simulated extinction spectrum of device 5 with the frequency-dependent noise.

SNR of photocurrent measurements

On the other hand, the SNR of the photocurrent measurement is given by the following equation: SNR=Power/NEP, where the power is the incident power of the QCL source on the device, as shown in the power spectrum in Supplementary Figure 27. The NEP is given by the responsivity and noise of the graphene detector, as shown in Supplementary Figure 2 and 10. Owing to the zero-bias operation, the noise of the detector is limited to Johnson noise, whose experimental value ranges from 1 to 4 pA. The typical photocurrent signals measured were approximately ∼60 nA and ∼500 nA for devices 2 and 3, respectively. This leads to a very high SNR that reaches a maximum value of 10^5. The SNR obtained using the previously mentioned equation is the same as that obtained by dividing the measured photocurrent signal by the current noise signal. We remark that we plotted the raw photocurrent data without any smoothing of the figures presented in the entire manuscript. Additionally, the SNR spectra are not limited to higher wavelengths, owing to the broadband absorption of graphene.

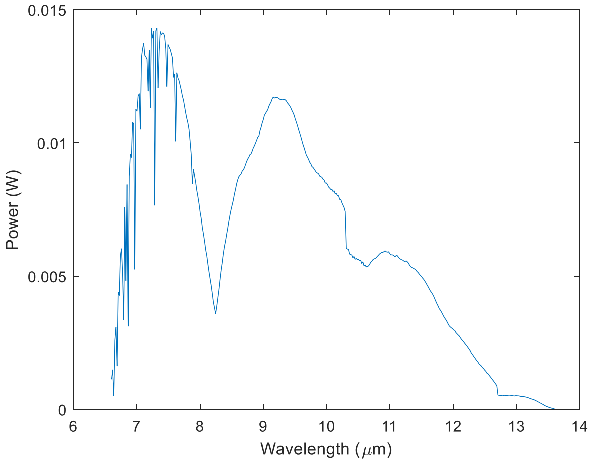

Supplementary Figure 27. Power spectrum of the QCL source.

The SNR is highest at the upper RB of the hBN, where the power and responsivity are the highest, as shown in Supplementary Figure 28. However, near the lower RB, the power drops significantly, and the responsivity is lower than that at the upper RB; therefore, we obtain a smaller SNR. We remark that for device 2, we obtain a smaller SNR in general owing to the higher resistance of the device (approximately 10 kΩ), which produces a smaller photocurrent compared to device 3 (resistance of 1 kΩ). Additionally, we observe that device 2 shows a small and flat SNR after 9 µm owing to the losses of SiO_2 optical phonons, as explained in the main text. However, in device, 3 we use a CaF_2 substrate, which is spectrally beneficial because it is transparent for this mid-infrared range. Therefore, device 3 exhibits a significantly higher SNR than device 2 in the lower RB range, as shown in Supplementary Figure 28b. We note that these high SNR values are still maintained even if we consider the peak power of the source to decrease by a factor of 1000 from 14 mW to 14 µW, we would obtain a maximum SNR value above 100 for device 3.

The SNR values reported in Figure 1b of the main text correspond to those extracted from the normalized photocurrent. We report the maximum SNR values of devices 2 and 3 in Figures 2a and 3a of the main text, respectively. For device 2 (3) we obtain a maximum SNR of 176 (1100), which corresponds to peak 6 (3), by considering the noise in the vicinity of the spectral range of the peak. We note that the photocurrent magnitude is significantly influenced by the doping of graphene. As a result, the normalized photocurrent amplitude is non-trivial because the maximum photocurrent values are achieved with the graphene doping near the CNP.[3] In contrast, when using FTIR, the optically active device containing polaritonic nanoresonators and the MCT detector operate as two independent systems. Therefore, the doping level of graphene does not impact the photoresponse behavior of the MCT detector.

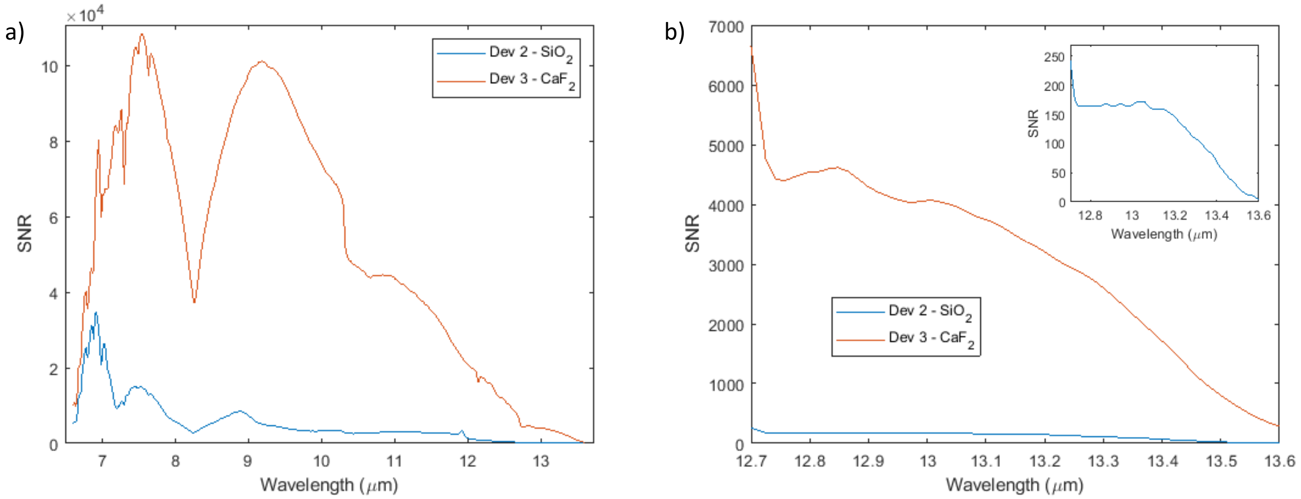

Supplementary Figure 28. **a)** SNR spectra of devices 2 and 3. In the legend, we indicate the substrate used for each device. **b)** Same as panel a, but for a zoomed region around the spectral range of the lower RB of hBN. The inset shows the SNR of device 2 in the same wavelength range.

SNR and noise comparison between FTIR and electrical spectroscopy

We compare other aspects of the devices, following the modified Figure 1b in the main text. We show in Supplementary Figure 29 how the SNR changes as a function of the total hBN thickness, period, width, and gap. The SNR of the FTIR increases with the period and width of the metallic nanorod. Similarly, when the total hBN thickness is decreased, the SNR increases; however, this represents a preliminary analysis, and further investigation is required.

By comparing the FTIR results with the theoretically calculated ones shown in Supplementary Figure 30, we observe that a larger period leads to a higher extinction, as shown experimentally. We notice that the simulated extinction values are significantly higher than the experimental values, particularly for device 4. This could be attributed to the size distribution of the width of the metallic nanorods and the gap between them, as explained in Supplementary Figure 4 and the main text, whose contribution can be even larger to be in better agreement with the experimental extinction values. The extinction noise increases by a factor of $\sim$2 in the wavelength range of 11-13 µm for all devices, as shown in Supplementary Figure 30c.

In the case of the photocurrent SNR, the signal is highly affected by the resistance value, which decreases the generated photocurrent, as shown in ref. 3. Therefore, the device with the highest resistance exhibits the lowest SNR, as shown in Supplementary Figure 31a. In addition, the estimated Johnson noise current obtained by considering the device resistance is very close to the measured experimental value, as exhibited in Supplementary Figure 31b.

The Supplementary Table II compares several parameters of the detector part of the electrical spectroscopy approach with those of traditional FTIR systems.

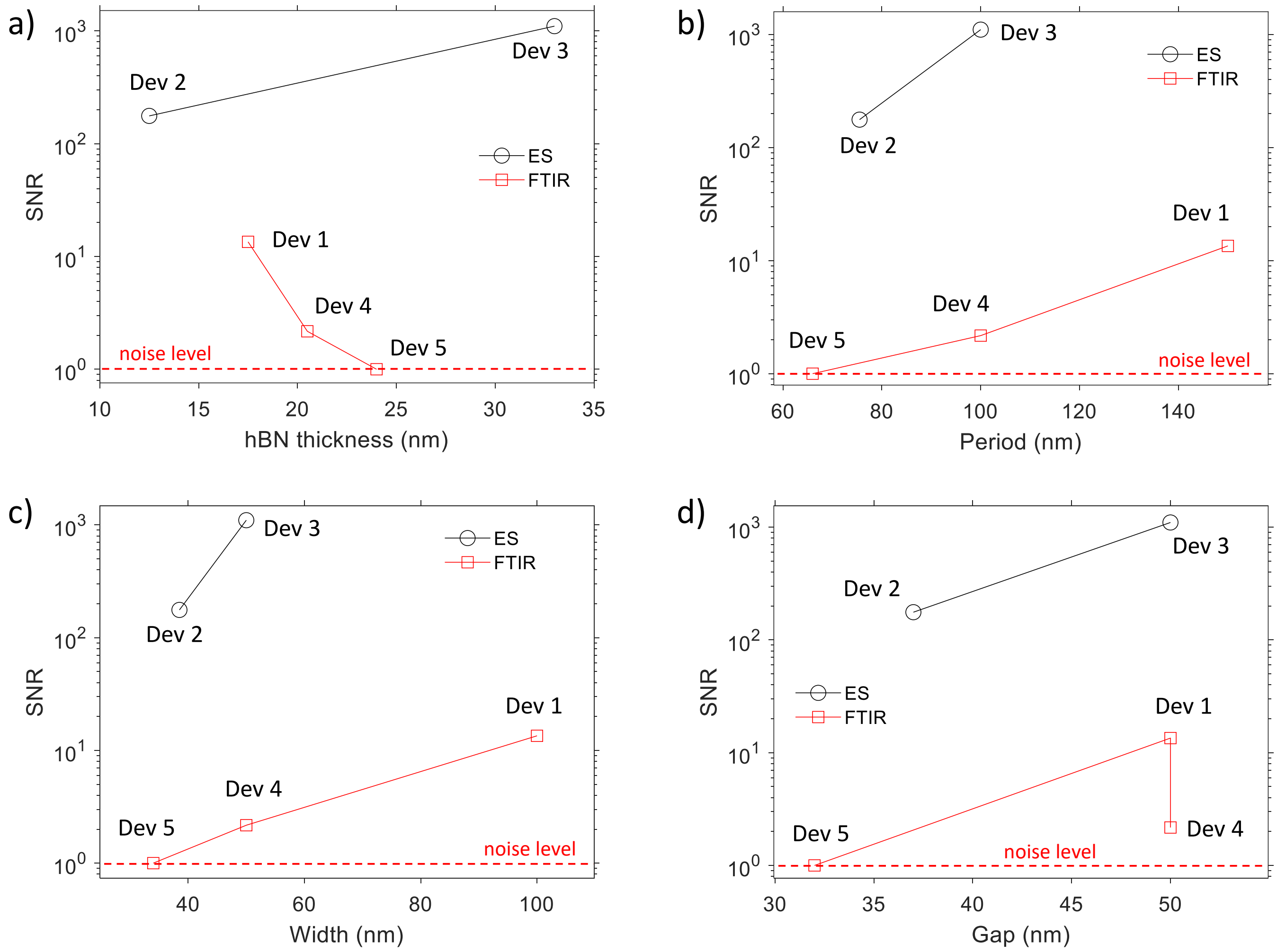

Supplementary Figure 29. SNR of the FTIR and electrical spectroscopy of the five devices as a function of **a)** hBN total thickness, **b)** period of the metallic nanorods, **c)** width of the nanorods, and **d)** gap between the nanorods. The red dashed line indicates the noise level that corresponds to an SNR of 1.

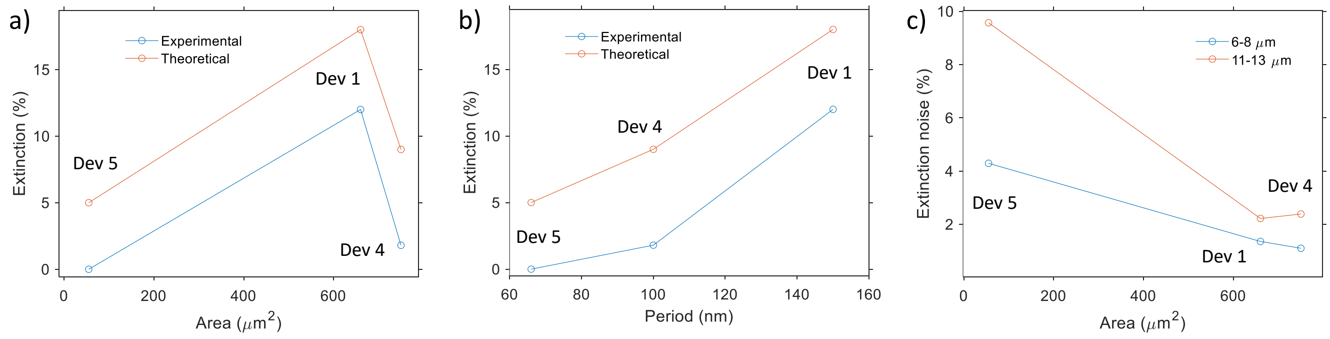

Supplementary Figure 30. Experimental and simulated extinction as a function of **a)** device area, and **b)** period of the metallic nanorods for devices 1, 4, and 5. **c)** Extinction noise (%) as a function of device area for devices 1, 4, and 5. Two main ranges are shown in the plot for 6- 8 µm and 11-13 µm. We notice that the extinction noise increases by a factor of ∼2 in the range of 11-13 µm.

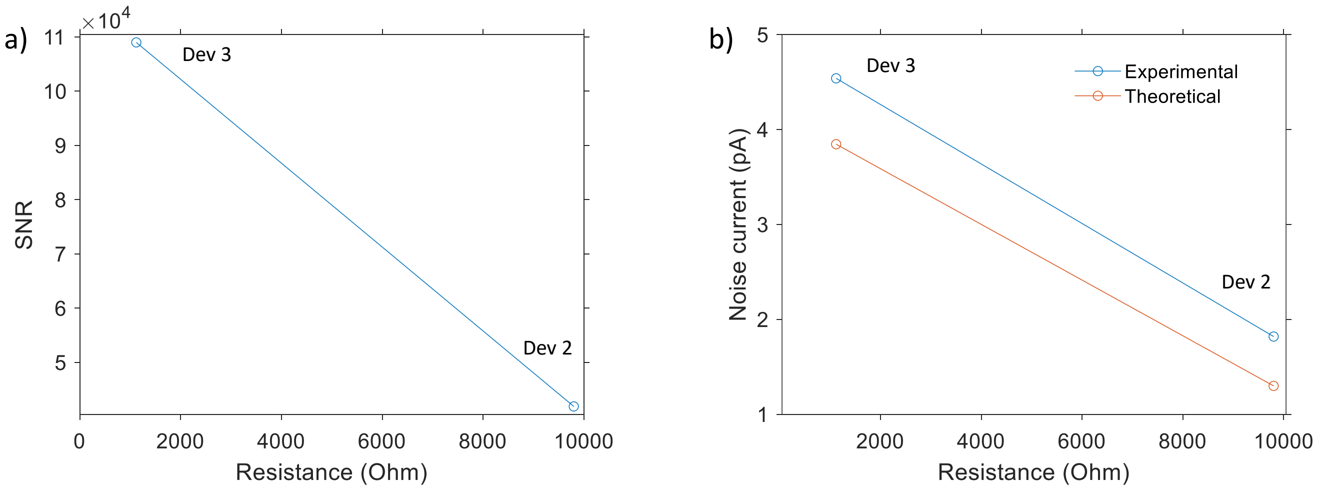

Supplementary Figure 31. SNR of photocurrent measurements as a function of the resistance of devices 2 and 3. **b)** Experimental and calculated noise current as a function of the resistance of devices 2 and 3.

**Parameter**	**FTIR spectroscopy**	**Electrical spectroscopy**
Voltage bias required	Yes	No
Cooling of the detector	Yes, with liquid nitrogen	No, room temperature operation
Smoothing of the data	Yes	No
Maximum SNR measured	13	1100
Minimum measurable sample area	660 μm^2	18 μm^2
CMOS compatibility	No	Yes
Size	Bulky	Compact
Fulfillment of SWaP	No	Yes
Operating wavelength	up to ∼12 µm	No wavelength cut-off

Supplementary Table II. FTIR and electrical spectroscopy comparison of the detector's main parameters.

SUPPLEMENTARY NOTE 3: SEMI-ANALYTICAL APPROACH TO THE MODELLING OF GRAPHENE PLASMONIC CRYSTAL.

We consider the structure, depicted in Fig. 32. The array of perfect electric conductor (PEC) bars is supposed to be periodic (with period D) along the direction of x-axis. The height and width of each PEC bar are d and $D-W$, respectively, thus each individual PEC bar occupies the spatial domain $d_b < z < d_b+d$, $lD+W/2 < z < (l+1)\,D-W/2$ (here l is the number of period). From the upper side the array of metal bars is covered by the layered structure, composed of the hBN bottom layer with thickness d_b (at region $0 < z < d_b$), graphene monolayer (arranged at $z=0$), and hBN top layer with thickness d_t (located at $-d_t < z < 0$). Note, that Fermi energy of graphene layer is supposed to be periodic function of the coordinate x, i.e. $E_F(x) = E_F(x+D)$. This hBN-graphene composite structure is supposed to be truncated by the semi-infinite vacuum/air, which occupies half-space $z < -d_t$. From bottom side the array of metal bars is deposited on top of another layered structure, which is composed of N capping layers, where each individual layer is characterized by thickness d_j [and occupies spatial domain $L_j < z < L_{j+1}$,where $L_j = d_t+d+\sum_{r=1}^{j-1} d_r$ is the coordinate of interface between $(j-1)$th and jth layers] and dielectric permeability $\varepsilon^{(j)}(\omega)$ [here $j = 1, ..., N$]. In its turn, this layered structure is deposited on top of semi-infinite substrate with dielectric function $\varepsilon^{(s)}(\omega)$, arranged at $z > L_{N+1}$. We also consider that incident wave with frequency ω falls normally on the above structure from vacuum/air side.

A. Solutions of Maxwell equations

Assuming electromagnetic field time-dependence as $\mathbf{E}, \mathbf{H} \sim \exp -i\omega t$, we represent Maxwell equations for p-polarized wave as

$$\frac{\partial E_x^{(\alpha)}}{\partial z} - \frac{\partial E_z^{(\alpha)}}{\partial x} = \frac{i\omega}{c} H_y^{(\alpha)}, \tag{1}$$

$$-\frac{\partial H_y^{(\alpha)}}{\partial z} = -\frac{i\omega}{c}\varepsilon_{xx}^{(\alpha)} E_x^{(\alpha)}, \tag{2}$$

$$\frac{\partial H_y^{(\alpha)}}{\partial x} = -\frac{i\omega}{c}\varepsilon_{zz}^{(\alpha)} E_z^{(\alpha)}. \tag{3}$$

where ω is wave cyclic frequency, c is the velocity of light in vacuum. The superscripts α correspond to the fields in different spatial domains, and will be specified below.

In the vacuum/air region $z < -d_t$ the solutions of Maxwell equation can be represented in the form of Fourier-Floquet series as

$$\begin{pmatrix} H_y^{(v)}(x,z) \\ E_x^{(v)}(x,z) \end{pmatrix} = \sum_{m=0}^{\infty} \hat{F}_m^{(v)} \begin{pmatrix} H_y^{(i)}\delta_{m,0}\exp\left[ip_m^{(v)}(z+d_t)\right] \\ H_{y||m}^{(r)}\exp\left[-ip_m^{(v)}(z+d_t)\right] \end{pmatrix} \times \cos\left[\frac{2\pi m}{D}x\right], \tag{4}$$

where

$$\hat{F}_m^{(v)} = \begin{pmatrix} 1 & 1 \\ \frac{cp_m^{(v)}}{\omega} & -\frac{cp_m^{(v)}}{\omega} \end{pmatrix} \tag{5}$$

is the field matrix,

$$p_m^{(v)} = \sqrt{\left(\frac{\omega}{c}\right)^2 - \left(\frac{2\pi m}{D}\right)^2}$$

is the out-of-plane wavevector component of mth harmonics, $H_y^{(i)}$ and $H_{y||m}^{(r)}$ are the amplitudes of the magnetic field of incident and reflected wave of mth harmonics. Since for normal incidence the in-plane wavevector component of incident wave is zero, the diffracted should be even function along x-axis. This fact allows an expansion with respect to cosine function in Eq. (4). Inside the semi-infinite substrate, $z > L_{N+1}$, the electromagnetic field can be expressed

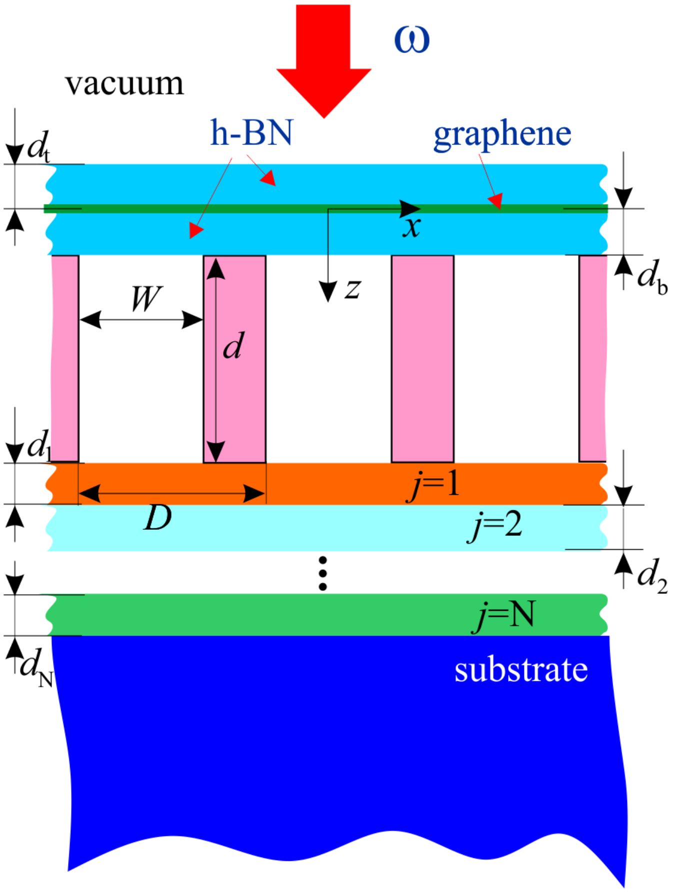

Supplementary Figure 32. Cross-sectional view of the studied geometry: diffraction grating made of PEC, arranged above layered substrate, and covered by the graphene layer, encapsulated between two hBN slabs. Figure schematic is not to scale.

as

$$\begin{pmatrix} H_y^{(s)}(x,z) \\ E_x^{(s)}(x,z) \end{pmatrix} = \sum_{m=0}^{\infty} \hat{F}_m^{(s)} \begin{pmatrix} H_{y||m}^{(s)} \exp\left[ip_m^{(s)}\left(z - L_{N+1}\right)\right] \\ 0 \end{pmatrix} \times \\ \times \cos\left[\frac{2\pi m}{D}x\right]. \tag{6}$$

Here

$$\hat{F}_m^{(s)} = \begin{pmatrix} 1 & 1 \\ \frac{cp_m^{(s)}}{\omega\varepsilon^{(s)}(\omega)} & -\frac{cp_m^{(s)}}{\omega\varepsilon^{(s)}(\omega)} \end{pmatrix}, \tag{7}$$

is the field matrix, $p_m^{(s)} = \sqrt{\left(\frac{\omega}{c}\right)^2 \varepsilon^{(s)}(\omega) - \left(\frac{2\pi m}{D}\right)^2}$ is the out-of-plane component of the wavevector of mth harmonics. In Eq. (6) zero in the second line means absence of the backward-propagating wave, and presence of the transmitted wave only (with amplitude of the magnetic field of mth harmonics $H_{y||m}^{(s)}$).

Inside the dielectric layers of finite thickness electromagnetic fields will be represented in the different manner – in form of the transfer-matrix

$$\hat{Q}_m^{(\alpha)}(z) = \begin{pmatrix} \cos\left[p_m^{(\alpha)}z\right] & \frac{i\omega\varepsilon_{xx}^{(\alpha)}(\omega)}{cp_m^{(\alpha)}} \sin\left[p_m^{(\alpha)}z\right] \\ \frac{icp_m^{(\alpha)}}{\omega\varepsilon_{xx}^{(\alpha)}(\omega)} \sin\left[p_m^{(\alpha)}z\right] & \cos\left[p_m^{(\alpha)}z\right] \end{pmatrix}, \tag{8}$$

which is written for the general case of anisotropic medium. For this case the out-of-plane component of wavevector is represented as$p_m^{(\alpha)} = \sqrt{\left(\frac{\omega}{c}\right)^2 \varepsilon_{xx}^{(\alpha)}(\omega) - \left(\frac{2\pi m}{D}\right)^2 \varepsilon_{xx}^{(\alpha)}(\omega)/\varepsilon_{zz}^{(\alpha)}(\omega)}$. To be more specific, total field in different layers

of structures will be represented in the following manner,

$$\begin{pmatrix} H_y^{(t)}(x,z) \\ E_x^{(t)}(x,z) \end{pmatrix} = \sum_{m=0}^{\infty} \hat{Q}_m^{(t)}(z+d_t) \times \times \begin{pmatrix} h_{y||m}^{(t)}(-d_t) \\ e_{x||m}^{(t)}(-d_t) \end{pmatrix} \cos\left[\frac{2\pi m}{D}x\right] \tag{9}$$

inside top hBN layer at $-d_t < z < 0$,

$$\begin{pmatrix} H_y^{(b)}(x,z) \\ E_x^{(b)}(x,z) \end{pmatrix} = \sum_{m=0}^{\infty} \hat{Q}_m^{(b)}(z) \times \times \begin{pmatrix} h_{y||m}^{(b)}(0) \\ e_{x||m}^{(b)}(0) \end{pmatrix} \cos\left[\frac{2\pi m}{D}x\right] \tag{10}$$

inside bottom hBN layer at $0 < z < d_b$, and

$$\begin{pmatrix} H_y^{(j)}(x,z) \\ E_x^{(j)}(x,z) \end{pmatrix} = \sum_{m=0}^{\infty} \hat{Q}_m^{(j)}(z-L_{j+1}) \times \times \begin{pmatrix} h_{y||m}^{(j)}(L_{j+1}) \\ e_{x||m}^{(j)}(L_{j+1}) \end{pmatrix} \cos\left[\frac{2\pi m}{D}x\right] \tag{11}$$

for jth layer in the bottom layered structure $L_j < z < L_{j+1}$. In the medium $d_b < z < d_b + d$, occupied by the PEC bars, the electromagnetic field can be represented as the superposition of waveguide modes inside the gaps. Thus, inside the spatial domain $lD - W/2 < x < lD + W/2$ (slits of width W) the tangential components of the electromagnetic field can be written as

$$\begin{pmatrix} H_{y||l}^{(g)}(x,z) \\ E_{x||l}^{(g)}(x,z) \end{pmatrix} = iW \sum_{n=0}^{\infty} \cos\left[\frac{2n\pi}{W}\left(x + \frac{W}{2} - lL\right)\right] \times \begin{pmatrix} \omega/c & \omega/c \\ \nu_n & -\nu_n \end{pmatrix} \begin{pmatrix} A_n^{(+,l)} \exp\left[i\nu_n (z-d_b)\right] \\ A_n^{(-,l)} \exp\left[-i\nu_n (z-d_b)\right] \end{pmatrix}, \tag{12}$$

where $\nu_n = \sqrt{\left(\frac{\omega}{c}\right)^2 - \left(\frac{2n\pi}{W}\right)^2}$, $A_n^{(\pm,l)}$ are the amplitudes of forward- and backward-propagating waves of the nth eigenmode in the lth gap.

B. Equation of the amplitudes of waveguide modes

As the boundary conditions at interfaces $z = -d_t$, and $z = L_j$ $(j = 2, ..., N+1)$ between homogeneous (in x-direction) dielectric layers without graphene we use continuity of tangential components of electromagnetic waves. At the same time at interface $z = 0$, containing graphene, tangential component of the electric field is also continuous across the interface, while the magnetic field tangential component is discontinuous across the interface owing the the presence of 2D currents in graphene $H_y^{(b)}(x,0) - H_y^{(t)}(x,0) = -(4\pi/c)\,\sigma^{(g)}(\omega,x)\,E_x^{(t)}(x,0)$, where

$$\sigma^{(g)}(\omega,x) = \frac{e^2}{\hbar^2\pi}\frac{E_F(x)}{\gamma(x) - i\omega} \tag{13}$$

is the Drude conductivity of graphene. The inverse relaxation time $\gamma(x)$ is also considered to be coordinate-dependent [as well as Fermi energy $E_F(x)$]. Being expressed through the electron mobility in graphene μ (which is considered to be constant), the inverse relaxation can be defined as

$$\gamma(x) = ev_F^2/\left[\mu E_F(x)\right] + v_F/D. \tag{14}$$

At surfaces of periodic grating, $z = d_b + d$ and $z = d_b + d$, the medium is non-homogeneous, here the imposed boundary conditions are of more complicated form: the continuity of electric and magnetic field tangential components at gaps

regions (between PEC bars, $lD - W/2 < x < lD + W/2$), and nullity of the electric field tangential component at the surfaces of PEC bars, $lD + W/2 < x < (l+1)\,D - W/2$.

Applying these boundary conditions, as well as Bloch theorem for normally incident wave $A_n^{(\pm,0)} \equiv A_n^{(\pm,l)}$, it is possible to obtain the system of coupled linear equations, which governs the amplitudes of the waveguide modes,

$$i\frac{\omega W}{c}\frac{1+\delta_{n',0}}{2}\left\{A_{n'}^{(+,0)} + A_{n'}^{(-,0)}\right\} -$$
$$i\frac{W^2}{D}\sum_{n=0}^{\infty}\nu_n\left\{A_n^{(+,0)} - A_n^{(-,0)}\right\}[\mathcal{P}_{n'}]^T\,\hat{\mathcal{F}}_{12}^{(v,tot)}\left[\hat{\mathcal{F}}_{22}^{(v,tot)}\right]^{-1}\hat{\mathcal{N}}\mathcal{P}_n = \tag{15}$$
$$[\mathcal{P}_{n'}]^T\left\{\hat{\mathcal{F}}_{11}^{(v,tot)} - \hat{\mathcal{F}}_{12}^{(v,tot)}\left[\hat{\mathcal{F}}_{22}^{(v,tot)}\right]^{-1}\hat{\mathcal{F}}_{21}^{(v,tot)}\right\}\mathcal{H}_y^{(i)},$$
$$i\frac{\omega W}{c}\frac{1+\delta_{n',0}}{2}\left\{A_{n'}^{(+,0)}\exp\left(i\nu_{n'}d\right) + A_{n'}^{(-,0)}\exp\left(-i\nu_{n'}d\right)\right\} - \tag{16}$$
$$i\frac{W^2}{D}\sum_{n=0}^{\infty}\nu_n\left\{A_n^{(+,0)}\exp\left(i\nu_n d\right) - A_n^{(-,0)}\exp\left(-i\nu_n d\right)\right\}[\mathcal{P}_{n'}]^T\,\hat{\mathcal{F}}_{11}^{(s,tot)}\left[\hat{\mathcal{F}}_{21}^{(s,tot)}\right]^{-1}\hat{\mathcal{N}}\mathcal{P}_n = 0.$$

Here $\mathcal{P}_n = \left(P_{n||0},\, P_{n||1},\, ...\right)^T$ is the column vector, whose elements are

$$P_{n||m} = \frac{DW}{\pi}\frac{m}{(mW)^2 - (nD)^2}\sin\left(\frac{\pi m W}{D}\right),$$

$\hat{\mathcal{N}}$ is the diagonal matrix with elements $\hat{\mathcal{N}}_{m',m} = (2-\delta_{m,0})\,\delta_{m,m'}$, $\mathcal{H}_y^{(i)} = \left(H_y^{(i)}, 0, 0, ...\right)^T$ is the incident wave's column vector. Also two total field matrices,

$$\hat{\mathcal{F}}^{(v,tot)} = \left[\hat{\mathcal{Q}}^{(b)}\left(d_b\right)\right]\hat{\mathcal{Q}}^{(g)}\left[\hat{\mathcal{Q}}^{(t)}\left(d_t\right)\right]\hat{\mathcal{F}}^{(v)} = \begin{pmatrix}\hat{\mathcal{F}}_{11}^{(v,tot)} & \hat{\mathcal{F}}_{12}^{(v,tot)}\\ \hat{\mathcal{F}}_{21}^{(v,tot)} & \hat{\mathcal{F}}_{22}^{(v,tot)}\end{pmatrix}, \tag{17}$$

$$\hat{\mathcal{F}}^{(s,tot)} = \left[\prod_{j=1}^{N}\hat{\mathcal{Q}}^{(j)}\left(-d_j\right)\right]\hat{\mathcal{F}}^{(s)} = \begin{pmatrix}\hat{\mathcal{F}}_{11}^{(s,tot)} & \hat{\mathcal{F}}_{12}^{(s,tot)}\\ \hat{\mathcal{F}}_{21}^{(s,tot)} & \hat{\mathcal{F}}_{22}^{(s,tot)}\end{pmatrix}, \tag{18}$$

are block matrices, composed of four submatrices and are obtained by multiplication of a series of other block matrices. Among them matrices

$$\hat{\mathcal{F}}^{(s)} = \begin{pmatrix}\hat{\mathcal{F}}_{11}^{(s)} & \hat{\mathcal{F}}_{12}^{(s)}\\ \hat{\mathcal{F}}_{21}^{(s)} & \hat{\mathcal{F}}_{22}^{(s)}\end{pmatrix},$$
$$\hat{\mathcal{F}}^{(v)} = \begin{pmatrix}\hat{\mathcal{F}}_{11}^{(v)} & \hat{\mathcal{F}}_{12}^{(v)}\\ \hat{\mathcal{F}}_{21}^{(v)} & \hat{\mathcal{F}}_{22}^{(v)}\end{pmatrix},$$
$$\hat{\mathcal{Q}}^{(j)}\left(-d_j\right) = \begin{pmatrix}\hat{\mathcal{Q}}_{11}^{(j)}\left(-d_j\right) & \hat{\mathcal{Q}}_{12}^{(j)}\left(-d_j\right)\\ \hat{\mathcal{Q}}_{21}^{(j)}\left(-d_j\right) & \hat{\mathcal{Q}}_{22}^{(j)}\left(-d_j\right)\end{pmatrix},$$
$$\hat{\mathcal{Q}}^{(\alpha)}\left(d_\alpha\right) = \begin{pmatrix}\hat{\mathcal{Q}}_{11}^{(\alpha)}\left(d_\alpha\right) & \hat{\mathcal{Q}}_{12}^{(\alpha)}\left(d_\alpha\right)\\ \hat{\mathcal{Q}}_{21}^{(\alpha)}\left(d_\alpha\right) & \hat{\mathcal{Q}}_{22}^{(\alpha)}\left(d_\alpha\right)\end{pmatrix}, \quad \alpha = t, b$$

are characterized by the fact that their submatrices are diagonal with elements

$$\hat{\mathcal{F}}_{11}^{(v)} = \hat{\mathcal{F}}_{12}^{(v)} = \hat{\mathcal{F}}_{11}^{(s)} = \hat{\mathcal{F}}_{12}^{(s)} = \hat{\mathcal{I}},$$

$$\left[\hat{\mathcal{F}}_{21}^{(v)}\right]_{m',m} = -\left[\hat{\mathcal{F}}_{22}^{(v)}\right]_{m',m} = \frac{cp_m^{(v)}}{\omega}\delta_{m',m},$$

$$\left[\hat{\mathcal{F}}_{21}^{(s)}\right]_{m',m} = -\left[\hat{\mathcal{F}}_{22}^{(s)}\right]_{m',m} = \frac{cp_m^{(s)}}{\omega\varepsilon^{(s)}(\omega)}\delta_{m',m},$$

$$\left[\hat{\mathcal{Q}}_{11}^{(j)}(-d_j)\right]_{m',m} = \cos\left[p_m^{(j)}d_j\right]\delta_{m',m},$$

$$\left[\hat{\mathcal{Q}}_{12}^{(j)}(-d_j)\right]_{m',m} = -\frac{i\omega\varepsilon^{(j)}(\omega)}{cp_m^{(j)}}\sin\left[p_m^{(j)}d_j\right]\delta_{m',m},$$

$$\left[\hat{\mathcal{Q}}_{21}^{(j)}(-d_j)\right]_{m',m} = -\frac{icp_m^{(j)}}{\omega\varepsilon^{(j)}(\omega)}\sin\left[p_m^{(j)}d_j\right]\delta_{m',m},$$

$$\left[\hat{\mathcal{Q}}_{22}^{(j)}(-d_j)\right]_{m',m} = \cos\left[p_m^{(j)}d_j\right]\delta_{m',m},$$

$$\left[\hat{\mathcal{Q}}_{11}^{(\alpha)}(-d_\alpha)\right]_{m',m} = \cos\left[p_m^{(\alpha)}d_\alpha\right]\delta_{m',m},$$

$$\left[\hat{\mathcal{Q}}_{12}^{(\alpha)}(d_\alpha)\right]_{m',m} = \frac{i\omega\varepsilon_{xx}^{(\alpha)}(\omega)}{cp_m^{(\alpha)}}\sin\left[p_m^{(\alpha)}d_\alpha\right]\delta_{m',m},$$

$$\left[\hat{\mathcal{Q}}_{21}^{(\alpha)}(d_\alpha)\right]_{m',m} = \frac{icp_m^{(\alpha)}}{\omega\varepsilon_{xx}^{(\alpha)}(\omega)}\sin\left[p_m^{(\alpha)}d_\alpha\right]\delta_{m',m},$$

$$\left[\hat{\mathcal{Q}}_{22}^{(\alpha)}(d_\alpha)\right]_{m',m} = \cos\left[p_m^{(\alpha)}d_\alpha\right]\delta_{m',m},$$

$\hat{\mathcal{I}}$ is the diagonal unity matrix. At the same time matrix

$$\hat{\mathcal{Q}}^{(g)} = \begin{pmatrix} \hat{\mathcal{I}} & -\frac{4\pi}{c}\hat{\Sigma} \\ 0 & \hat{\mathcal{I}} \end{pmatrix},$$

possesses one non-diagonal submatrix $\hat{\Sigma}$, which appears owing to the nonuniformity of graphene's conductivity across one period of the structure. The elements of this submatrix are

$$\left[\hat{\Sigma}\right]_{m',m} = \frac{1}{2}\left[\frac{1+\delta_{m'+m,0}}{1+\delta_{m',0}}\sigma_{m+m'}^{(g)}(\omega) + \frac{1+\delta_{m'-m,0}}{1+\delta_{m',0}}\sigma_{|m-m'|}^{(g)}(\omega)\right],$$

where

$$\sigma_m^{(g)}(\omega) = \tfrac{4}{D(1+\delta_{m,0})}\int_0^{D/2}dx\,\sigma^{(g)}(\omega,x)\cos\left(\tfrac{2\pi m}{D}x\right)$$

is the Fourier component of graphene's conductivity such that

$$\sigma^{(g)}(\omega,x) = \sum_{m=0}^{\infty}\sigma_m^{(g)}(\omega)\cos\left(\frac{2\pi m}{D}x\right).$$

After solving Eqs.(15) and (16), from amplitudes $A_n^{(\pm,0)}$ it is possible to obtain amplitudes of harmonics of reflected, $\mathcal{H}_y^{(r)} = \left(H_{y||0}^{(r)}, H_{y||1}^{(r)}, H_{y||2}^{(r)}, ...\right)^T$, and transmitted, $\mathcal{H}_y^{(s)} = \left(H_{y||0}^{(s)}, H_{y||1}^{(s)}, H_{y||2}^{(s)}, ...\right)$, waves in the form

$$\mathcal{H}_y^{(r)} = i\frac{W^2}{D}\sum_{n=0}^{\infty}\nu_n\left\{A_n^{(+,l)} - A_n^{(-,l)}\right\}\left[\hat{\mathcal{F}}_{22}^{(v,tot)}\right]^{-1}\hat{\mathcal{N}}\mathcal{P}_n - \left[\hat{\mathcal{F}}_{22}^{(v,tot)}\right]^{-1}\hat{\mathcal{F}}_{21}^{(v,tot)}\mathcal{H}_y^{(i)}. \quad (19)$$

$$\mathcal{H}_y^{(s)} = i\frac{W^2}{D}\sum_{n=0}^{\infty}\nu_n\left\{A_n^{(+,l)}\exp(i\nu_n d) - A_n^{(-,l)}\exp(-i\nu_n d)\right\}\left[\hat{\mathcal{F}}_{21}^{(s,tot)}\right]^{-1}\hat{\mathcal{N}}\mathcal{P}_n. \quad (20)$$

C. Reflectance, transmittance and absorbance

The reflectance (transmittance) coefficients can be obtained as the ratio between z-component of Poynting vector of reflected (transmitted) wave's relation and that of incident wave,

$$R=-\frac{\mathrm{Re}\left\{\left[\mathcal{H}_y^{(r)}\right]^{\dagger}\hat{\mathcal{F}}_{22}^{(v)}\hat{\mathcal{N}}^{-1}\mathcal{H}_y^{(r)}\right\}}{\left|H_y^{(i)}\right|^2}, \tag{21}$$

$$T=\frac{\mathrm{Re}\left\{\left[\mathcal{H}_y^{(s)}\right]^{\dagger}\hat{\mathcal{F}}_{21}^{(s)}\hat{\mathcal{N}}^{-1}\mathcal{H}_y^{(s)}\right\}}{\left|H_y^{(i)}\right|^2}. \tag{22}$$

In Eq. (21) for reflection coefficient sign minus appears owing the the propagation of reflected wave in negative direction of axis z, while in Eq. (22) for transmittance coefficient sign plus is accounted to the fact, that transmitted wave propagates in the positive direction of z-axis. The total absorption A of the structure can be calculated as

$$A=1-R-T,$$

while absorption by the graphene only, A_g, can be represented as

$$A_g=\frac{\mathrm{Re}\left\{\left[\mathcal{E}_x^{(t)}\left(0\right)\right]^{\dagger}\hat{\mathcal{N}}^{-1}\hat{\Sigma}\mathcal{E}_x^{(t)}\left(0\right)\right\}}{\left|H_y^{(i)}\right|^2},$$

where $\mathcal{E}_x^{(t)}\left(0\right)=\left(e_{x||0}^{(t)}\left(0\right), e_{x||1}^{(t)}\left(0\right), ...\right)^T$ is the column vector of electric field harmonic amplitudes on the graphene.

I. PHONON-PLASMON-POLARITONS EIGENMODES.

The dispersion relation of the phonon-plasmon-polaritons in the graphene-hBN hybrid structure can be calculated via transfer-matrix method. For the eigenmode problem field matrices in the vacuum and substrate can be obtained by formal substitution of $2\pi m/D=k_x$ into respective field matrices for the excitation problem [see Eqs. (22) and (7)], thus obtaining

$$\hat{F}_{k_x}^{(v)}=\begin{pmatrix}1 & 1\\ \frac{cp_{k_x}^{(v)}}{\omega} & -\frac{cp_{k_x}^{(v)}}{\omega}\end{pmatrix} \tag{23}$$

$$\hat{F}_{k_x}^{(s)}=\begin{pmatrix}1 & 1\\ \frac{cp_{k_x}^{(s)}}{\omega\varepsilon^{(s)}(\omega)} & -\frac{cp_{k_x}^{(s)}}{\omega\varepsilon^{(s)}(\omega)}\end{pmatrix}, \tag{24}$$

where $p_{k_x}^{(v)}=\sqrt{\left(\frac{\omega}{c}\right)^2-k_x^2}$, $p_{k_x}^{(s)}=\sqrt{\left(\frac{\omega}{c}\right)^2\varepsilon^{(s)}\left(\omega\right)-k_x^2}$ are out-of-plane component of wavevectors inside the vacuum and substrate, respectively. Using field matrices 23and 24, fields at the hBN-vacuum and substrate interfaces can be represented as

$$\begin{pmatrix}h_{y||k_x}^{(v)}\left(-d_t\right)\\ e_{x||k_x}^{(v)}\left(-d_t\right)\end{pmatrix}=\hat{F}_{k_x}^{(v)}\begin{pmatrix}0\\ H_{y||k_x}^{(r)}\end{pmatrix}, \tag{25}$$

$$\begin{pmatrix}h_{y||k_x}^{(s)}\left(L_{N+1}\right)\\ e_{x||k_x}^{(s)}\left(L_{N+1}\right)\end{pmatrix}=\hat{F}_{k_x}^{(s)}\begin{pmatrix}H_{y||k_x}^{(s)}\\ 0\end{pmatrix}. \tag{26}$$

Zero at the first row of Eq. 25 describes absence of incident wave, propagating in the positive direction of z-axis – a situation, typical for eigenmodes [compare with Eq. 4], while zero in second row of Eq. 26 has the same sense, as that in Eq. 7. At the same time fields at opposite sides of finite-thickness layers can be related via transfer-matrices, namely

$$\begin{pmatrix}h_{y||k_x}^{(t)}\left(0\right)\\ e_{x||k_x}^{(t)}\left(0\right)\end{pmatrix}=\hat{Q}_{k_x}^{(t)}\left(d_t\right)\begin{pmatrix}h_{y||k_x}^{(t)}\left(-d_t\right)\\ e_{x||k_x}^{(t)}\left(-d_t\right)\end{pmatrix} \tag{27}$$

inside top hBN layer at $-d_t < z < 0$,

$$\begin{pmatrix} h^{(b)}_{y||k_x}(d_b) \\ e^{(b)}_{x||k_x}(d_b) \end{pmatrix} = \hat{Q}^{(b)}_{k_x}(d_b) \begin{pmatrix} h^{(b)}_{y||k_x}(0) \\ e^{(b)}_{x||k_x}(0) \end{pmatrix} \tag{28}$$

inside bottom hBN layer at $0 < z < d_b$, and

$$\begin{pmatrix} h^{(j)}_{y||k_x}(L_j) \\ e^{(j)}_{x||k_x}(L_j) \end{pmatrix} = \hat{Q}^{(j)}_{k_x}(-d_j) \begin{pmatrix} h^{(j)}_{y||k_x}(L_{j+1}) \\ e^{(j)}_{x||k_x}(L_{j+1}) \end{pmatrix} \tag{29}$$

for jth layer in the bottom layered atructure $L_j < z < L_{j+1}$. In the above equations the transfer-matrices are obtained from Eq. 8 by the substitution of $2\pi m/D = k_x$,

$$\hat{Q}^{(\alpha)}_{k_x}(z) = \begin{pmatrix} \cos\left[p^{(\alpha)}_{k_x} z\right] & \frac{i\omega\varepsilon^{(\alpha)}_{xx}(\omega)}{cp^{(\alpha)}_{k_x}} \sin\left[p^{(\alpha)}_{k_x} z\right] \\ \frac{icp^{(\alpha)}_{k_x}}{\omega\varepsilon^{(\alpha)}_{xx}(\omega)} \sin\left[p^{(\alpha)}_{k_x} z\right] & \cos\left[p^{(\alpha)}_{k_x} z\right] \end{pmatrix}, \tag{30}$$
$$p^{(\alpha)}_{k_x} = \sqrt{\left(\frac{\omega}{c}\right)^2 \varepsilon^{(\alpha)}_{xx}(\omega) - k_x^2 \varepsilon^{(\alpha)}_{xx}(\omega)/\varepsilon^{(\alpha)}_{zz}(\omega)}.$$

Boundary conditions at interfaces without graphene are continuity of electric and magnetic field tangential component, while boundary conditions across the graphene (at interface $z = 0$) can be expressed in matrix form

$$\begin{pmatrix} h^{(b)}_{y||k_x}(0) \\ e^{(b)}_{x||k_x}(0) \end{pmatrix} = \hat{Q}^{(g)} \begin{pmatrix} h^{(t)}_{y||k_x}(0) \\ e^{(t)}_{x||k_x}(0) \end{pmatrix}, \tag{31}$$
$$\hat{Q}^{(g)} = \begin{pmatrix} 1 & -\frac{4\pi}{c}\overline{\sigma^{(g)}(\omega)} \\ 0 & 1 \end{pmatrix},$$

and average graphene's conductivity $\overline{\sigma^{(g)}(\omega)}$ will be different for the acoustic and graphene modes.

For the acoustic mode we use the equivalent scheme, depicted in Fig. 33(a) – a graphene layer, cladded between two hBN layers, and deposited on top of PEC. This structure is equivalent to one, depicted in Fig. 32, but when $W = 0$. Notice, that layers at $z > d + d_b$ are screened by PEC. The dispersion relation can be obtained by consequent multiplication of matrices in Eqs. 25, 27, 31, and 28, thus obtaining expression for the electromagnetic fields on the surface of PEC as

$$\begin{pmatrix} h^{(b)}_{y||k_x}(d_b) \\ e^{(b)}_{x||k_x}(d_b) \end{pmatrix} = \hat{F}^{(v,tot)}_{k_x} \begin{pmatrix} 0 \\ H^{(r)}_{y||k_x} \end{pmatrix}, \tag{32}$$

where $\hat{F}^{(v,tot)}_{k_x} = \left[\hat{Q}^{(b)}_{k_x}(d_b)\right] \hat{Q}^{(g)} \left[\hat{Q}^{(t)}_{k_x}(d_t)\right] \hat{F}^{(v)}_{k_x}$ is the total field matrix [similar to one in Eq. 17]. Here the expression for the graphene's average conductivity in boundary condition matrix 31 is defined as

$$\overline{\sigma^{(g)}(\omega)} = \frac{1}{W} \int_{-W/2}^{W/2} \sigma^{(g)}(\omega, x)\, dx,$$

i.e. averaged in the region above single PEC bar. The boundary condition on the PEC is zero tangential component of electric field on PEC's surface, i.e. $e^{(b)}_{x||k_x}(d_b) = 0$. Being substituted into 32, it gives the dispersion relation for the acoustuc mode in the form

$$\left[\hat{F}^{(v,tot)}_{k_x}\right]_{22} = 0.$$

To obtain the dispersion relation of graphene modes the equivalent scheme can be obtained from that in Fig. 32 by putting $W = D$. In this case the periodic structure is substituted by vacuum of finite thickness d, as it is shown in Fig. 33(b). The fields at the finite vacuum bottom boundary $z = d_b + d$ can be obtained by consecutive multiplication of matrices in Eqs. 26 and 29, thus giving

$$\begin{pmatrix} h^{(1)}_{y||k_x}(d_b + d) \\ e^{(1)}_{x||k_x}(d_b + d) \end{pmatrix} = \hat{F}^{(s,tot)}_{k_x} \begin{pmatrix} H^{(s)}_{y||k_x} \\ 0 \end{pmatrix}, \tag{33}$$

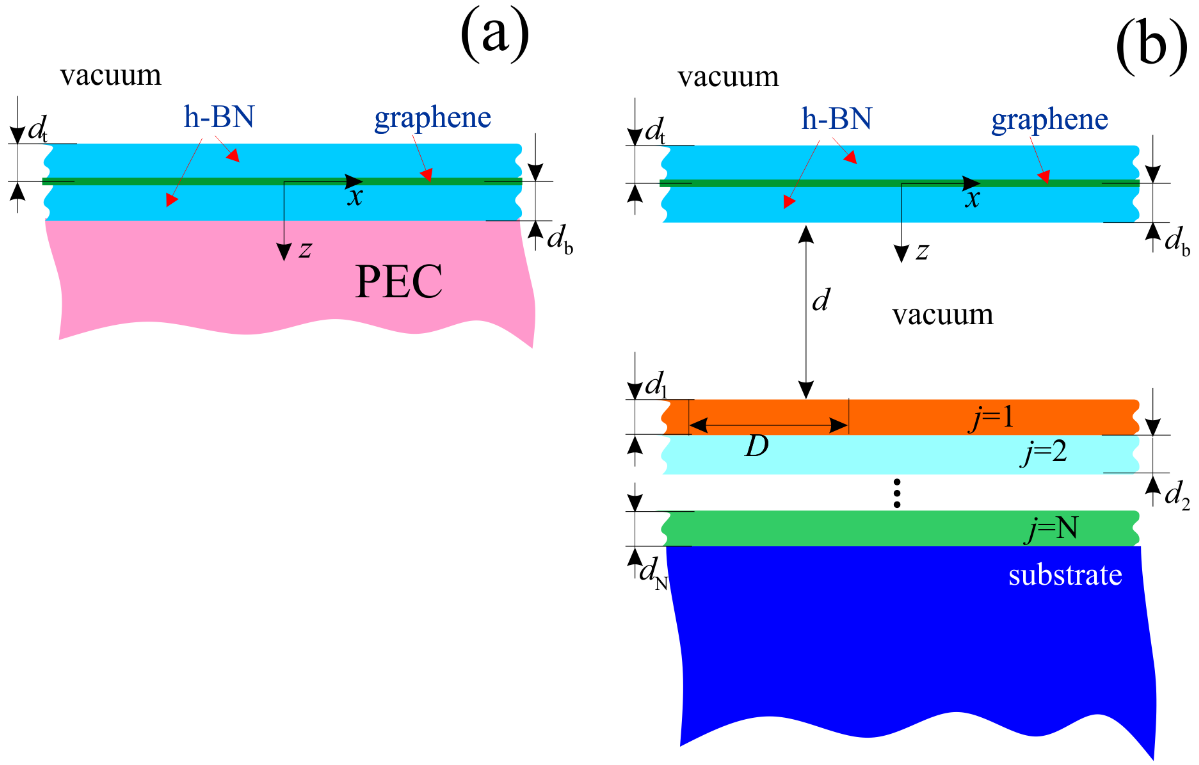

Supplementary Figure 33. Equivalent schemes for the dispersion relation of acoustic [panel (a)] and graphene modes [panel (b)].

where $\hat{F}^{(s,tot)}_{k_x} = \left[\prod_{j=1}^{N} \hat{Q}^{(j)}_{k_x}(-d_j)\right] \hat{F}^{(s)}_{k_x}$ is the total field matrix, whose structure is similar to that in Eq. 18. At the same time, fields at both sides of finite thickness vacuum can be related via transfer matrix as

$$\begin{pmatrix} h^{(1)}_{y||k_x}(d_b+d) \\ e^{(1)}_{x||k_x}(d_b+d) \end{pmatrix} = \hat{Q}^{(v)}_{k_x}(d) \begin{pmatrix} h^{(b)}_{y||k_x}(d_b) \\ e^{(b)}_{x||k_x}(d_b) \end{pmatrix}, \tag{34}$$

where $\varepsilon^{(v)}_{xx}(\omega) = \varepsilon^{(v)}_{zz}(\omega) = 1$. Combining Eqs. 32, 33, and 34, we have

$$\hat{F}^{(s,tot)}_{k_x} \begin{pmatrix} H^{(s)}_{y||k_x} \\ 0 \end{pmatrix} = \hat{Q}^{(v)}_{k_x}(d)\, \hat{F}^{(v,tot)}_{k_x} \begin{pmatrix} 0 \\ H^{(r)}_{y||k_x} \end{pmatrix},$$

from which the dispersion relation for graphene mode can be obtained in the form

$$\left[\left(\hat{F}^{(s,tot)}_{k_x}\right)^{-1} \hat{Q}^{(v)}_{k_x}(d)\, \hat{F}^{(v,tot)}_{k_x}\right]_{22} = 0. \tag{35}$$

Notice, that the averaging of graphene's conductivity in boundary condition matrix 31 [which in its turn is included into matrix $\hat{F}^{(v,tot)}_{k_x}$ in dispersion relation 35] is performed over the region above the gap between PEC bars, i.e.

$$\overline{\sigma^{(g)}(\omega)} = \frac{1}{D-W} \int\limits_{W/2}^{D-W/2} \sigma^{(g)}(\omega, x)\, dx.$$

SUPPLEMENTARY REFERENCES

[1] Iranzo, D. A. et al. Probing the ultimate plasmon confinement limits with a van der waals heterostructure. *Science* **360**, 291–295 (2018).

[2] Epstein, I. et al. Far-field excitation of single graphene plasmon cavities with ultracompressed mode volumes. *Science* **368**, 1219–1223 (2020).

[3] Castilla, S. et al. Plasmonic antenna coupling to hyperbolic phonon-polaritons for sensitive and fast mid-infrared photodetection with graphene. *Nature Communications* **11**, 4872 (2020).

[4] Castilla, S. et al. Fast and Sensitive Terahertz Detection Using an Antenna-Integrated Graphene pn Junction. *Nano Letters* **19**, 2765–2773 (2019).

[5] Dai, S. et al. Graphene on hexagonal boron nitride as a tunable hyperbolic metamaterial. *Nature Nanotechnology* **10**, 682–686 (2015).

[6] Woessner, A. et al. Highly confined low-loss plasmons in graphene–boron nitride heterostructures. *Nature Mater.* **14**, 421–425 (2015).

[7] Caldwell, J. D. et al. Low-loss, infrared and terahertz nanophotonics using surface phonon polaritons. *Nanophotonics* **4**, 44–68 (2015).

[8] Tielrooij, K. J. et al. Out-of-plane heat transfer in van der Waals stacks through electron-hyperbolic phonon coupling. *Nature Nanotechnology* **13**, 41–46 (2018).

[9] Viti, L. et al. Thermoelectric graphene photodetectors with sub-nanosecond response times at terahertz frequencies. *Nanophotonics* **10**, 89–98 (2021).

[10] Vangelidis, I. et al. Unbiased Plasmonic-Assisted Integrated Graphene Photodetectors. *ACS Photonics* **9**, 1992–2007 (2022).

[11] Lee, I. H. et al. Image polaritons in boron nitride for extreme polariton confinement with low losses. *Nature Communications* **11** (2020).

[12] Brar, V. W., Jang, M. S., Sherrott, M., Lopez, J. J. & Atwater, H. A. Highly confined tunable mid-infrared plasmonics in graphene nanoresonators. *Nano Letters* **13**, 2541–2547 (2013).

[13] Brar, V. W. et al. Hybrid Surface-Phonon-Plasmon Polariton Modes in Graphene/Monolayer h-BN Heterostructures. *Nano Letters* **14**, 3876–3880 (2014).

[14] ThermoFisher-Scientific. *Ftir microscopy: How experimental decisions affect the signal-to-noise ratio.* **available at http://www.thermofisher.com/RaptIR** (2024).

[15] Matsumoto, M. & Nishimura, T. Mersenne twister: a 623-dimensionally equidistributed uniform pseudo-random number generator. (1998). 8, 1, 3–30.
